# Deep learning-based detection of construction waste in complex scenarios with an improved lightweight algorithm

**DOI:** 10.1371/journal.pone.0356914

**Published:** 2026-09-18

**Authors:** YiZhong Yang, YeXue Li, ShengWei Li, MaoHu Tao, Xuzhi Chen

**Affiliations:** 1 School of Automotive and Traffic Engineering, Hubei University of Arts and Science, Xiangyang, China; 2 School of Civil Engineering and Architecture, Hubei University of Arts and Science, Xiangyang, China; 3 Hubei Key Laboratory of Vehicle-Infrastructure Collaboration and Traffic Control, Xiangyang, China; 4 School of Mathematics and Statistics, Hubei University of Arts and Science, Xiangyang, China; 5 Hubei Gongjian Hanjiang Engineering Co., Ltd., Xiangyang, China; Zhejiang University, CHINA

## Abstract

To tackle the challenges in construction waste detection under complex scenarios—such as insufficient recognition accuracy, significant feature variations among identical waste categories, limited publicly available CDW datasets, and excessive computational resource consumption that hinders real-time performance—this paper proposes a novel improved algorithm, GTS-YOLO. Built on YOLOv11, GTS-YOLO achieves model lightweighting by optimizing the C3K2 module in the backbone network, enhances detection accuracy effectively through integrating spatial attention mechanisms between the backbone and neck, and redesigns the detection head with reference to the task alignment principle to better handle object detection of construction waste under occlusion and deformation. On our self-constructed 10-category dataset, compared with YOLOv11n, GTS-YOLO increases mAP50 by 3.24% to 67.47%, improves Precision by 3.1%, and reduces the parameter count by 19.4%, In addition, the model size is only 4.23 MB, which is approximately 20.6% smaller than YOLOv11n, demonstrating the effectiveness of the proposed lightweight design.achieving an effective balance between accuracy and efficiency.We have also validated the model performance on public datasets, demonstrating its generalization ability across diverse scenarios.

## 1 Introduction

With the accelerated advancement of urbanization, the construction industry has played an increasingly prominent role in contributing to the national economy. Concomitantly, the issue of construction waste has become more pronounced. In 2020, an estimated 10 gigatons of construction waste were generated worldwide [1]. It is estimated that the European Union (EU) generates approximately 450 million tons (MT) of construction and demolition waste annually, of which only 28% is recycled, with the remaining 72% disposed of in landfill [2]. As the country with the highest global solid waste emissions, China generates approximately 1 billion tons of new solid waste annually, with historical stockpiles reaching 60–70 billion tons. Among these, the resource utilization rate of construction waste remains below 50% [3]. Construction and demolition waste (CDW) not only occupies a large amount of land but also causes pollution to soil, water bodies, and air, severely restricting environmental carrying capacity and resource circular utilization. Therefore, how to efficiently and rapidly handle the increasing construction waste has become a rather pressing issue. In the traditional construction waste recycling industry, manual sorting methods are characterized by low efficiency and poor accuracy. Additionally, debris and dust generated from the crushing of concrete, bricks, and plaster layers may cause damage to workers” respiratory systems. To address the growing disposal demand, optimize processing workflows, enhance recycling rates, and protect worker health, automated sorting of construction waste has become imperative.

Currently, some construction waste recycling companies employ magnetic separators and eddy current separators to extract magnetic and non-magnetic metals, respectively. They also utilize gravity and hydraulic washing to recover sand, silt, and light impurities by leveraging density differences for water-based stratification. Although the above-mentioned processes can partially replace manual labor, the preliminary screening stage still relies on workers for visual inspection and intervention or classification of abnormal large items, foreign matter contamination, and other situations. Meanwhile, with the rapid development of computer vision and deep learning technologies, automated sorting solutions based on deep models have stood out among numerous approaches.

In deep learning, object detection technology has achieved a leapfrog development from shallow models based on manual features to end-to-end deep learning models, significantly enhancing the efficient and rapid recognition capabilities for tiny, occluded, and diversified targets. As early as 2015, multi-stage detection frameworks based on the R-CNN (Region-based Convolutional Neural Network) series demonstrated exceptional detection accuracy on general datasets such as PASCAL Visual Object Classes (VOC) and Common Objects in Context (COCO). Subsequently, one-stage models like YOLO (You Only Look Once) and Single Shot Detector (SSD) advanced detection speed to practical levels through unified feature extraction and regression pipelines, laying the foundation for real-time applications. Building on this foundation, construction waste detection, as a crucial subfield of object detection, has started to attract substantial research attention. In single-stage models, Zhou et al. [4] achieved a mAP@0.5 of 0.948 for construction waste detection by integrating CBAM (Convolutional Block Attention Module), SimSPPF (Simplified Spatial Pyramid Pooling-Fast), and multi-scale detection into YOLOv5. This breakthrough demonstrates the effectiveness of hybrid architecture design in addressing the unique challenges of construction waste scenarios, such as complex object occlusion, irregular shapes, and diverse material compositions. Demetriou et al. [5] conducted a comprehensive performance comparison of one-stage and multi-stage models for construction waste detection, revealing that YOLOv7 demonstrates superior accuracy and inference speed. Notably, YOLOv7 proved particularly suitable for scenarios involving stacked and agglutinated waste objects, outperforming other architectures in handling complex occlusion and material adhesion. In the realm of multi-stage models, Zhang et al. [6] proposed a two-stage algorithm named $W^{2}R$, which integrates fine-grained recognition with high-level classification. The first stage identifies 13 subcategories, while the second stage categorizes wastes into four major classes. This framework achieved an overall accuracy of 94.71% on automated sorting machines, demonstrating its effectiveness in bridging detailed material identification and practical waste management workflows. Pang et al. [7] proposed a two-stage construction waste detection framework, where the model first identifies bounding boxes for waste items and then classifies them into one of six major categories. This framework enhances detection performance by optimizing data utilization strategies, effectively reducing overfitting and bias in complex waste sorting scenarios. Li et al. [8] proposed MCCNN for detection and classification of municipal solid waste images. MCCNN integrates three subnetworks—DSSD, YOLOv4, and Faster-RCNN—via a hierarchical feature fusion strategy. Experimental results on a benchmark municipal solid waste image dataset show that the model achieved a 10% improvement in average detection accuracy compared to single-model baselines, demonstrating the effectiveness of cross-model feature complementarity in handling complex waste material variations. Alrayes et al. [9] proposed a hybrid architecture integrating Vision Transformer (ViT) and Multi-Layer Hybrid Convolutional Neural Network (MLH-CNN), achieving 95.8% accuracy on the TrashNet dataset. This framework leverages ViT”s global contextual modeling capability and MLH-CNN”s local feature extraction efficiency, outperforming traditional CNNs by 5.6% in waste classification tasks. Qi et al. [10] leveraged the parallel computing advantages of Transformers to employ the DETR (Detection Transformer) model for small object detection. Experiments on waste detection validated the model’s accuracy in identifying small objects. Vineet Prasad et al. [11] proposed the ShARP-WasteSeg framework for instance segmentation, which leverages shape-aware approaches to optimize mask boundary quality for stacked construction and demolition waste. Testing on a CDW dataset validated the framework”s effectiveness in enhancing boundary precision for occluded and overlapping waste objects. Yang et al. [12] proposed the FE-YOLO lightweight model, which integrates the ECA (Efficient Channel Attention) mechanism and Faster_c2f module into YOLOv8. On a self-built construction waste dataset, FE-YOLO achieved a 3% improvement in mAP@50 while reducing parameter count by 12% and FLOPs by 13%, demonstrating its effectiveness in balancing detection accuracy and computational efficiency. Zheng et al. [13] proposed a lightweight model based on MobileNetV2, integrating the Focus module and knowledge distillation for substantial parameter compression. This approach achieved 92% accuracy on the TrashNet dataset, demonstrating effective balance between model efficiency and classification performance. Shad et al. [14] proposed LD-YOLOv9s, which enhances YOLOv9s through three key modifications: replacing convolutional layers in the backbone with DynConvLayer, integrating the SDConv-ADown module, and adopting MPD-IoU in place of CIoU. On the LD-2024 dataset, this approach achieved a 6.3% improvement in mean average precision (mAP) compared to the baseline YOLOv9s, demonstrating the effectiveness of dynamic convolution and optimized loss functions in waste detection scenarios. Casao et al. [15] introduced the first industrial-grade multimodal dataset combining Hyperspectral Imaging (HSI) and RGB data, validating the advantages of multimodal segmentation in plastic sorting. This dataset provides a comprehensive solution for material identification in complex industrial scenarios by integrating spectral and spatial information, addressing the limitations of single-modality methods in handling contaminated or composite plastics. Lu et al. [16] fused visual (VGG16) and acoustic (1D CNN) data, improving mixed waste classification accuracy through feature-level multimodal fusion. Sirimewan et al. [17] proposed the CDW-Seg dataset as a benchmark dataset for construction waste classification and segmentation in the direction of improving datasets and annotations. Yudin et al. [18] released the Warp dataset, which contains over 10,000 samples of 28 recyclable material classes in industrial scenario images with overlaps, poor lighting, or severe distortions. The dataset enables high-precision instance segmentation via the weakly-supervised segmentation method H-YC. Majchrowska et al. [19] improved the accuracy of multi-class waste classification to 75% via a semi-supervised training strategy, leveraging unlabeled data to enhance model performance. Demetriou et al. [20] created a dataset named CODD as a benchmark for construction waste detection and proposed a baseline model based on YOLOv8.

However, despite the numerous advances in detection accuracy, lightweight design, and multimodal fusion achieved by existing studies, several critical issues still need to be urgently addressed. Firstly, publicly available waste datasets predominantly focus on municipal solid waste or urban environmental waste, with very few large-scale datasets dedicated to CDW. Moreover, some CDW datasets are only suitable for instance segmentation, while benchmark datasets for object detection are even rarer. The limited variety of these datasets fails to cover all common CDW types, rendering models difficult to accurately recognize waste in real construction site scenarios. Secondly, many methods introduce complex network architectures or multi-stage processes in pursuit of accuracy, significantly increasing computational load and deployment costs. This hinders their application on edge devices or mobile field terminals. Furthermore, some studies remain confined to single improvement directions and lack systematic ablation comparisons, making it difficult to objectively evaluate the independent contributions of mechanisms, cascaded subnetworks, and other components to overall performance. Finally, although multimodal approaches enhance robustness, they impose higher requirements on sensors, annotations, and data preprocessing, further escalating the practical engineering challenges.

To address the trade-off challenges among parameter count, detection accuracy, and generalization capability in existing methods, this paper makes the following improvements:

(1) Construction Waste Dataset Construction: We collected and annotated approximately 500 images of stacked construction waste on-site, covering ten common construction waste types: wood, steel bars, concrete blocks, concrete fragments, bricks, scaffolding, asphalt chunks, small stones, foam, and tiles. This provides rich, real-world distribution samples for model training in construction site scenarios.
(2) GTS-YOLO Model Based on YOLOv11: We propose the GTS-YOLO model, which reduces parameters while improving detection accuracy.
(3) Lightweight Feature Extraction Module: By integrating an improved GDconv into the C3K2 module of YOLOv11 and replacing the traditional bottleneck module with the GDModule structure, we develop the GD_C3K2 module. This module effectively reduces parameter count and FLOPs while maintaining accurate extraction of basic features from samples.
(4) SimAM Attention Mechanism: Connected at the junction of the backbone and neck networks, this attention mechanism focuses on tiny targets (e.g., broken bricks, small stones) and suppresses background noise without extra parameters, ensuring that features transmitted to the neck are more discriminative and robust.
(5) Task-Aligned Detection Head (Tahead): Aimed at the multi-category and multi-form characteristics of construction waste, Tahead adopts parameter sharing and task alignment strategies for classification and regression branches, achieving precise localization and classification of various wastes while balancing real-time performance and accuracy.

## 2 Related research

### 2.1 Overview of YOLOv11 model

**Fig 1:**
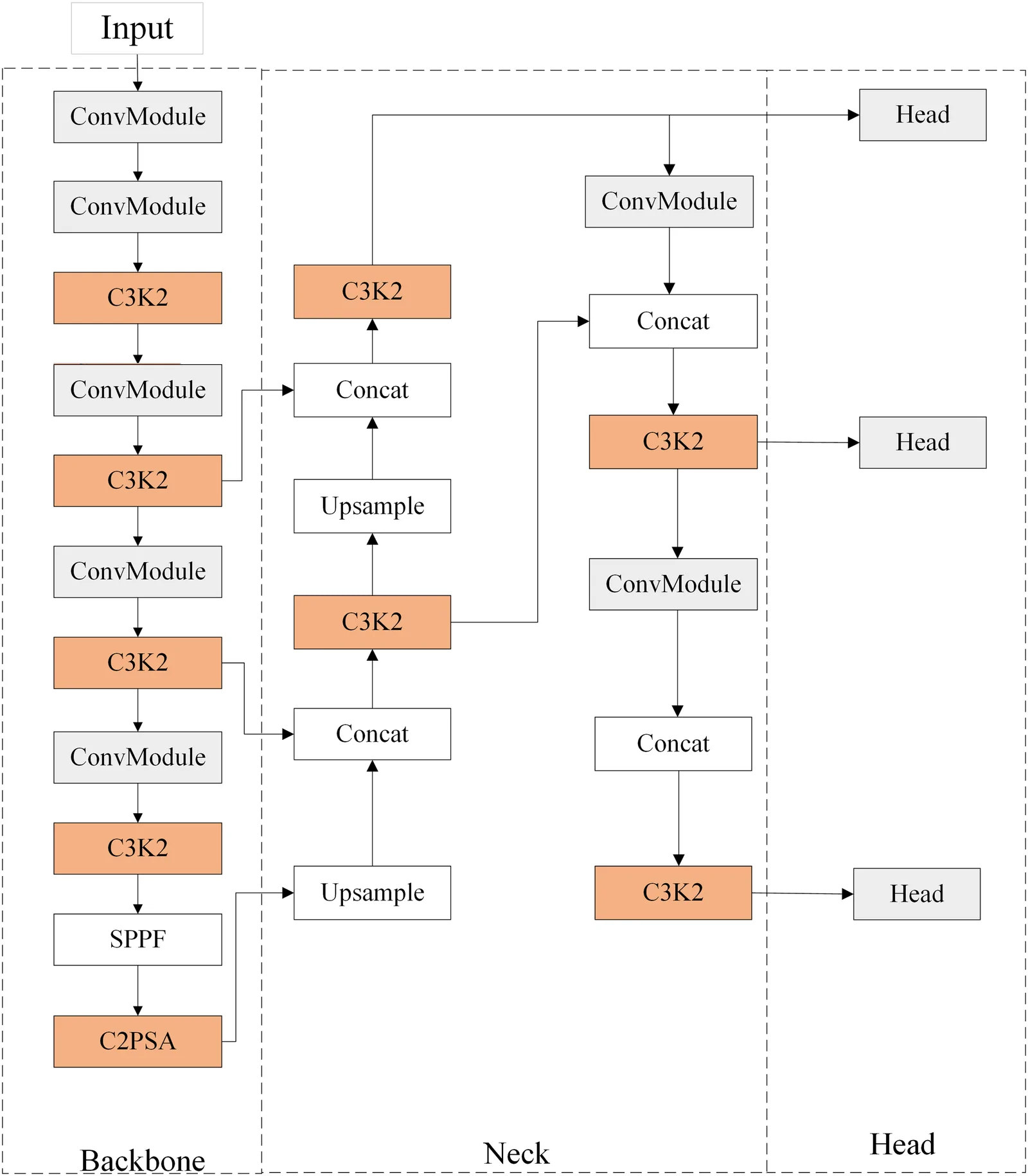
Structure of YOLOv11 model.

Developed by Ultralytics in October 2024, YOLOv11 introduces several systematic optimizations in network architecture design to significantly outperform preceding versions (such as YOLOv8, YOLOv9, and YOLOv10) in both accuracy and computational efficiency. Its comprehensive architecture consists of three core components—backbone, neck, and head—as illustrated in Fig 1.and its key architectural enhancements are detailed and analyzed in literature [21]. In this study, the lightweight variant YOLOv11n is selected as the baseline model.

Within the framework, each component is engineered to enhance feature representation while controlling parametric growth. Specifically, in the backbone, parameter-efficient C3K2 blocks are integrated with C2PSA attention modules to balance computational cost, allowing the network to focus on highly informative regions by capturing spatial contextual dependencies. In the neck, C3K2 blocks substitute the traditional C2f modules; this modification strengthens multi-scale feature fusion and improves the interaction between shallow and deep semantic features, thereby maintaining precise localization performance even under complex scenarios with dense objects and scale variations. Finally, the detection head cooperatively integrates C3K2 modules, CBS (Convolution–BatchNorm–SiLU) layers, and convolutional prediction layers to simultaneously output bounding box coordinates, objectness scores, and category probabilities.

In terms of computational efficiency, YOLOv11n demonstrates high deployment viability, requiring only 6.4 GFLOPs. Across numerous benchmark datasets, the YOLOv11 series [22] effectively balances precision and speed when detecting objects of varying sizes, providing an exceptionally robust and reliable foundation for our proposed construction waste detection task. The YOLOv11 families [23] have fully demonstrated comprehensive advantages.

### 2.2 Deformable convolution

**Fig 2:**
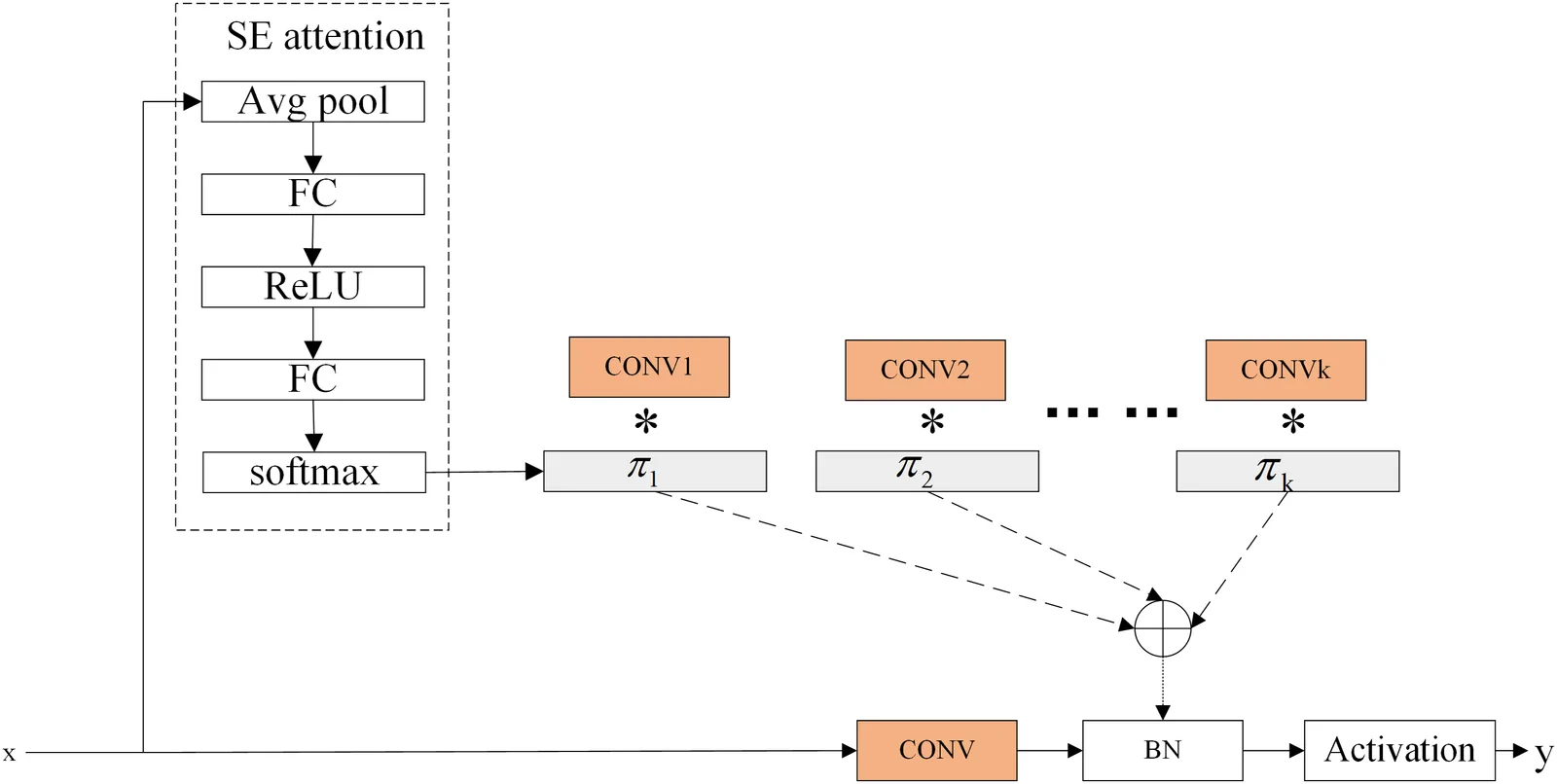
Schematic diagram of Dynamic Convolution.

Dynamic Convolution and Deformable Convolutional Networks (DCN), as innovative deformable convolution techniques in the field of deep learning, have extensive applications in computer vision. Dynamic Convolution can dynamically generate convolution kernel parameters based on input features. In image classification scenarios, it can generate adaptive convolution kernels according to different image contents, thereby enhancing the ability to capture diverse image features and improving classification accuracy. The schematic diagram of Dynamic Convolution is shown in Fig 2. Dynamic Convolution also has K kernels. Following the classic design in CNN, the authors followed the dynamic convolution with BatchNorm and ReLU. The authors used a lightweight squeeze-and-excitation mechanism to extract attention weights. While SENet assigns attention mechanisms to channels [24], dynamic convolution uses it to assign attention mechanisms to convolution kernels. Due to the small size of the kernels, the kernel integration process is computationally efficient. The authors validated the effectiveness of the proposed method on the ImageNet dataset. Baseline models included MobileNetV2, V3, ResNet, etc. The number of kernels K in dynamic convolution was set to 4, and the attention weight normalization factor τ was set to 30. It was verified that dynamic convolution can consistently improve performance while only increasing the computational cost by 4% [25].

**Fig 3:**
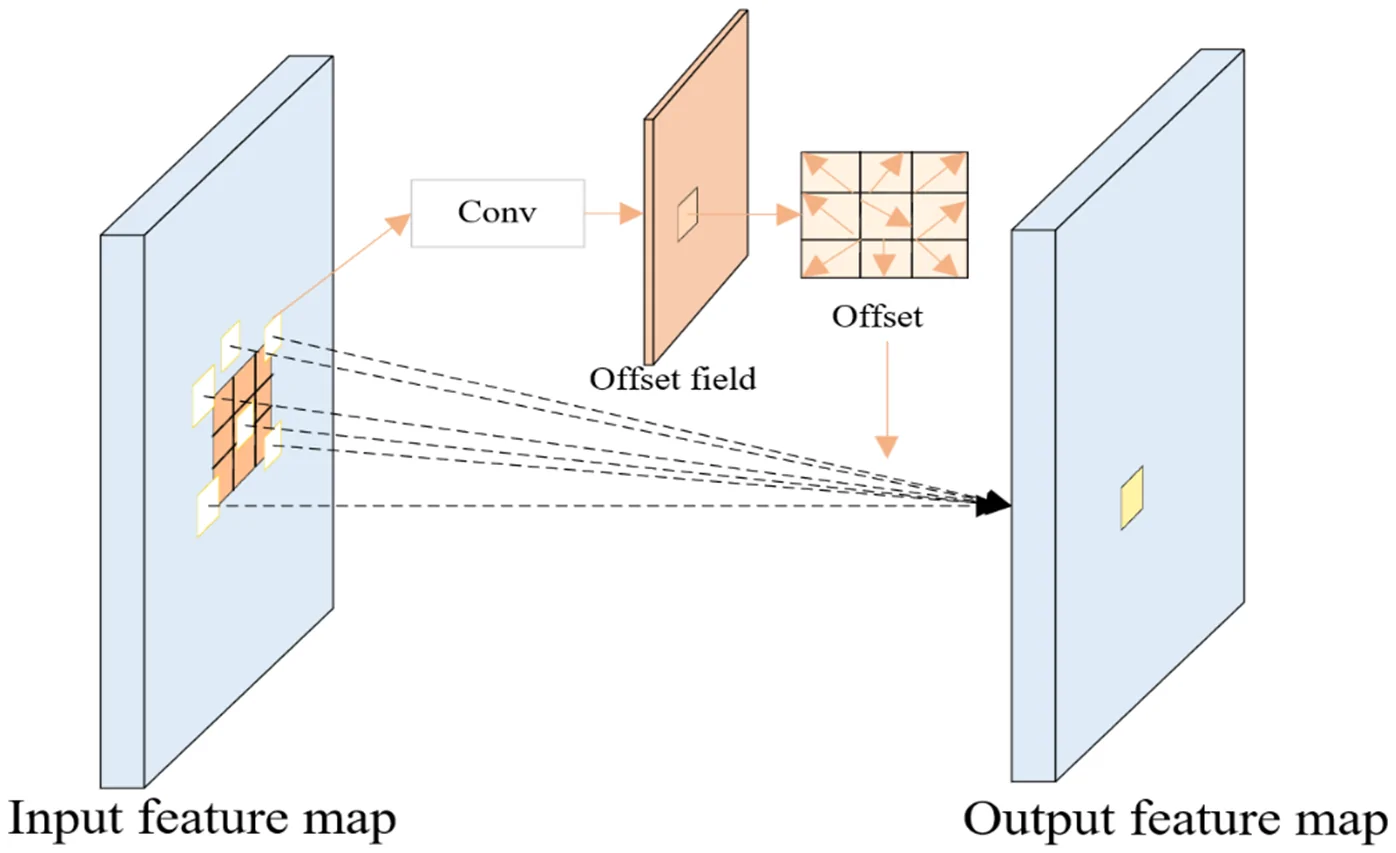
Schematic diagram of Deformable Convolutional Networks (DCN).

The schematic diagram of Deformable Convolutional Networks (DCN) is shown in Fig 3. DCN v1 adaptively adjusts the receptive field shape of convolution kernels by learning the offsets of sampling points. In the field of object detection, it can better capture features of objects with varying poses and irregular shapes, thereby improving detection accuracy. The Deformable Conv operation does not change the computational process of traditional convolution but introduces learnable parameters to the regions where convolution is applied.

For each output position, the sampling points are allowed to spread into non-uniform shapes. The offsets are derived by applying convolution to the original feature map. Given an input feature map of size w×h×c, a convolution operation is first performed to generate an offset field of size w×h×2c. Here, *w* and *h* remain the same as those of the original feature map. Each value in the offset field represents the displacement of the corresponding position in the input feature map, with separate values for the *x* and *y* directions, hence the channel number 2*c*. Since the offsets obtained through convolution are often floating-point numbers, bilinear interpolation is employed to compute the feature values at the offset positions. During the learning process of offsets, gradients are backpropagated through the bilinear interpolation operation [26]. Building upon DCN v1, DCN v2 introduces an additional modulation weight, which increases the model’s flexibility. This allows the model to assign zero weights to unimportant sampling points, effectively modulating the spatial distribution and mutual influence of samples through learned feature amplitudes [27].

Although Dynamic Convolution and Deformable Convolution both introduce adaptive mechanisms into the traditional convolution operation, their optimization strategies focus on different aspects of the convolution process. Dynamic Convolution improves feature representation by dynamically generating or aggregating convolution kernel weights according to the input features, allowing the convolution filters to adapt to different visual patterns in the data. In contrast, Deformable Convolution enhances spatial modeling capability by learning offsets for sampling locations, enabling the convolution operation to adjust its receptive field to better align with objects exhibiting geometric variations or irregular shapes. Therefore, Dynamic Convolution mainly performs content-adaptive kernel modulation, whereas Deformable Convolution achieves spatially adaptive feature sampling. This distinction clarifies their respective mechanisms and highlights their complementary roles in improving feature representation and spatial alignment in convolutional neural networks.

### 2.3 Attention mechanisms

Attention mechanisms originate from the selective focus mechanism of the human visual perception system, which prioritizes important information. They were first introduced to the field of deep learning to address the vanishing gradient problem and long-range dependency issues encountered by Recurrent Neural Networks (RNNs) when processing lengthy sequential data. Bahdanau et al. [28] pioneered this technology by applying it to machine translation tasks. They achieved effective information exchange between the encoder and decoder by dynamically assigning weights to different input positions, thereby laying the foundation for this technology. In the realm of deep learning, attention mechanisms have become a key technology for enhancing model performance. These mechanisms can be broadly categorized as follows:

1. Global Feature Interaction: Examples include BiLevelRouting, which employs a two-level routing mechanism to facilitate cross-channel and cross-region feature interaction. It is well-suited for tasks requiring long-range dependency modeling, such as semantic segmentation and object detection. Its advantage lies in its ability to capture global contextual information, while its drawback is relatively high computational complexity [29].
2. Relationship Modeling: For instance, Triplet leverages triplet contrastive learning to explore relationships among samples. It excels at enhancing feature discriminability but heavily relies on hard sample mining [30].
3. Lightweight and Efficient: Take ECA as an example, which implements channel attention through one-dimensional convolution. With extremely low computational overhead, it is ideal for mobile-models. It features a small number of parameters and high speed, yet its representational capacity is limited [31].

Additionally, several other attention mechanisms are designed for specific scenarios and offer unique advantages:

SegNext: Region-based attention that heightens focus on specific regions in segmentation tasks [32].
Dual attention: Deals with tasks involving directional features, such as image super-resolution [33].
Window Attention: Used in Vision Transformers to reduce computational complexity [34].
CAFM: Strengthens feature interaction in multimodal tasks [35].
AGCA: Increases attention to important channels [36].

In practical applications, an appropriate attention mechanism should be selected according to task requirements, computational resources, and model architecture to strike a balance between performance improvement and computational cost.

### 2.4 Task alignment

In the field of object detection, two critical challenges currently exist. Firstly, the classification and localization tasks are typically handled by independent branches, resulting in a lack of interaction between them. This often leads to inconsistencies during prediction, such as high-scoring predictions with inaccurate positions or precise localizations receiving low confidence scores. Secondly, common sample assignment strategies, whether used in geometry-based anchor-free object detectors or IoU-based anchor-based detectors, are task-agnostic and cannot simultaneously meet the requirements of accurate classification and localization. To address these issues, C Feng et al. [37] proposed the Task-Aligned One-Stage Object Detector (TOOD) and designed the Task-aligned Head (T-head). The principle behind TOOD is to enhance the interaction between classification and localization by computing their interactive features and leverage the Task-Aligned Predictor (TAP) for prediction, thereby achieving more accurate object detection.

**Fig 4:**
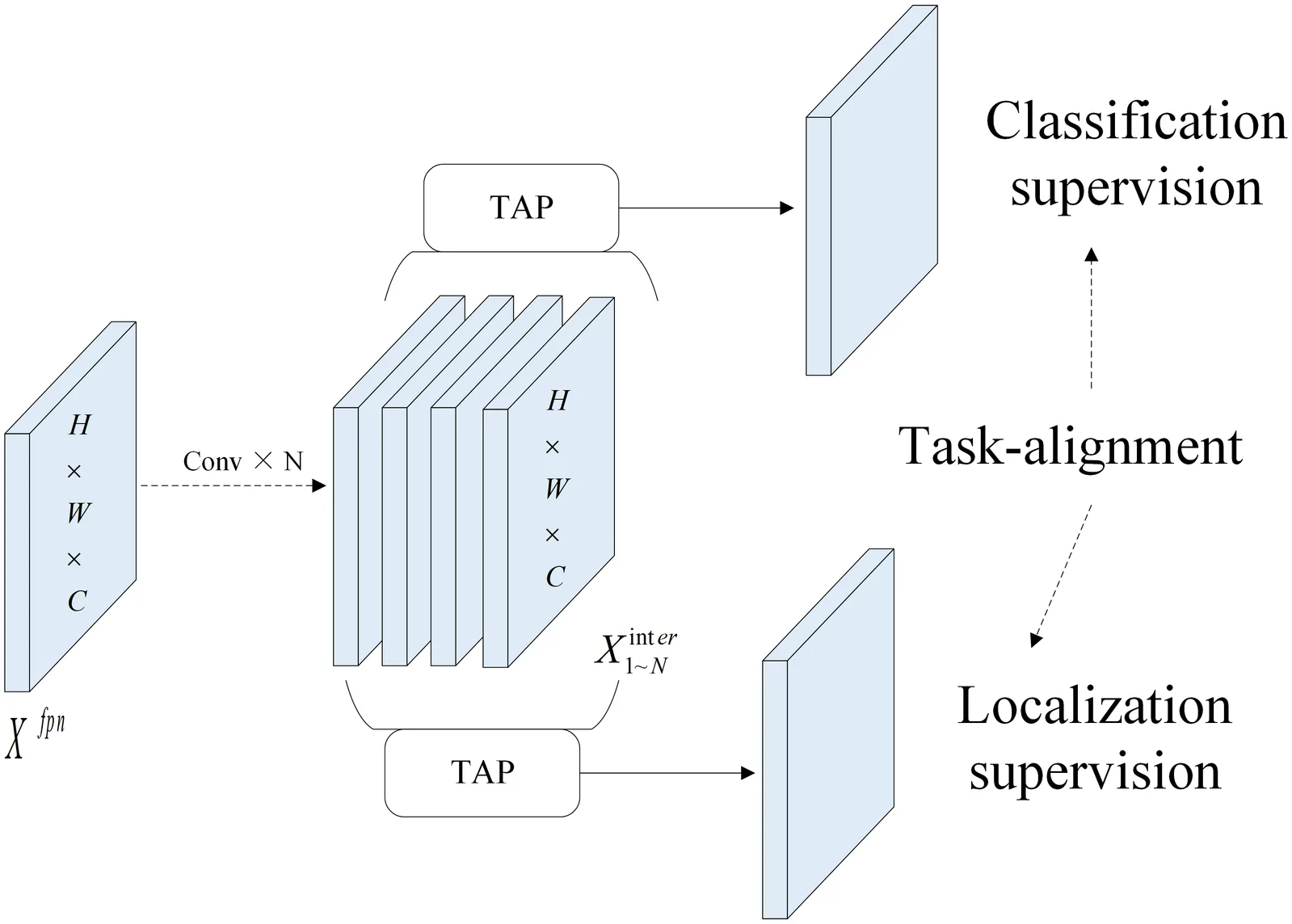
Task-aligned head structure.

As shown in Fig 4, first, after the FPN features are input, they are processed by multiple convolutional layers in the feature extractor. The interactive features $X_{k}^{inter}$ output by each layer are concatenated into comprehensive features. At this time, when entering the TAP, the global interactive feature $x^{inter}$ first undergoes dimension reduction through the fully connected layer *fc*_1_, introduces nonlinearity via the δ activation function, then undergoes dimension increase through the fully connected layer *fc*_2_, and finally generates a normalized weight vector $w=\sigma \left(fc_{2}\left(\delta \left(fc_{1}\left(x^{inter}\right)\right)\right)\right)$ by the activation function. This weight performs weighted summation on the interactive features $X_{k}^{inter}$ of each layer as $X_{k}^{task}=w_{k}\cdot X_{k}^{inter}$, achieving the separation of task-specific features. Subsequently, in the classification branch, the cls_decomp module combines the $x^{inter}$ after global average pooling to weight the comprehensive features, generates the original classification score P through two layers of convolution, and simultaneously learns the alignment matrix M from the interactive features, and obtains equation 1 through geometric average operation.


$$\begin{array}{c} P^{\text{align }}=\sqrt{P\times M} \end{array}$$ (1)

Completes the alignment of classification scores and feature quality; The localization branch processes features in the same way, the convolution generates regression distances, and after scaling and exponential operations, the original localization box B is obtained according to the anchor type. Meanwhile, the two-dimensional offset O is learned from the interactive features, and coordinate offset is performed on B through deformable convolution to correct the position of the localization box. The formula is shown in in Eq. (2).


$$\begin{array}{c} B^{\text{align }}(i,j,c)=B(i+O(i,j,2c),j+O(i,j,2c+1),c) \end{array}$$ (2)

In this process, the core mechanism of TAP is reflected in: dynamically generating the weight w through cross-layer task interaction features, enabling the classification and localization tasks to respectively focus on their optimal feature subsets. Meanwhile, with the help of M and O, it realizes the explicit alignment of scores and coordinates. Finally, in the three stages of feature extraction, task decomposition, and result adjustment, it solves the problem of conflict between classification and localization features in traditional detection.

## 3 Methodology

Model structure optimization and feature enhancement mechanisms play a decisive role in detection accuracy and efficiency. Detection accuracy and parameter quantity are core elements for efficient construction waste detection. In this research, to achieve high-precision and lightweight construction waste detection, we propose the GTS-YOLO model. In the backbone network part of the model, by introducing GDconv, the number of model parameters and the amount of computation are effectively reduced. At the connection between the backbone network and the neck network, the SimAM attention mechanism is integrated. Without increasing the number of parameters, it successfully overcomes the problems of insufficient capture of small-target features and interference from background noise. Based on the characteristics of the construction waste detection task, and referring to the idea of task alignment, a task-adapted detection head is redesigned. This detection head realizes the integration of efficient detection performance and a parameter fusion structure through parameter sharing and task alignment strategies. GTS-YOLO provides the possibility of both accuracy and practicality for the real-time detection model of construction waste.

### 3.1 GD_C3K2 module

Convolutional neural networks face a critical trade-off between computational efficiency and feature expression capability. Traditional convolution operations, while effective in extracting image features, have high computational complexity, which limits the deployment of models in resource-constrained environments. The GhostModule proposed by GhostNet divides feature maps into primary features and derived features, significantly reducing the computational load while maintaining feature expression capability, thus providing an effective solution for lightweight network design [38]. However, the static convolution kernels used in traditional GhostModule lack flexibility when dealing with complex scenarios and are difficult to adapt to changes in different input patterns. DynamicConv generates adaptive convolution kernel weights by introducing an attention mechanism, which can dynamically adjust the convolution process according to input content and significantly enhance the model’s ability to represent complex patterns. Nevertheless, the direct application of DynamicConv will bring additional computational overhead. Therefore, it is necessary to redesign GhostModule to maintain the computational efficiency of GhostModule while enhancing its feature expression capability. To this end, we propose the GDModule. By replacing traditional convolution with DynamicConv in the GhostModule structure, linear representation is achieved through depth-wise separable convolution. Furthermore, the Bottleneck structure in C3K2 is replaced with GDModule to improve C3K2, thereby achieving an effective balance between computational efficiency and feature expression capability.

**Fig 5:**
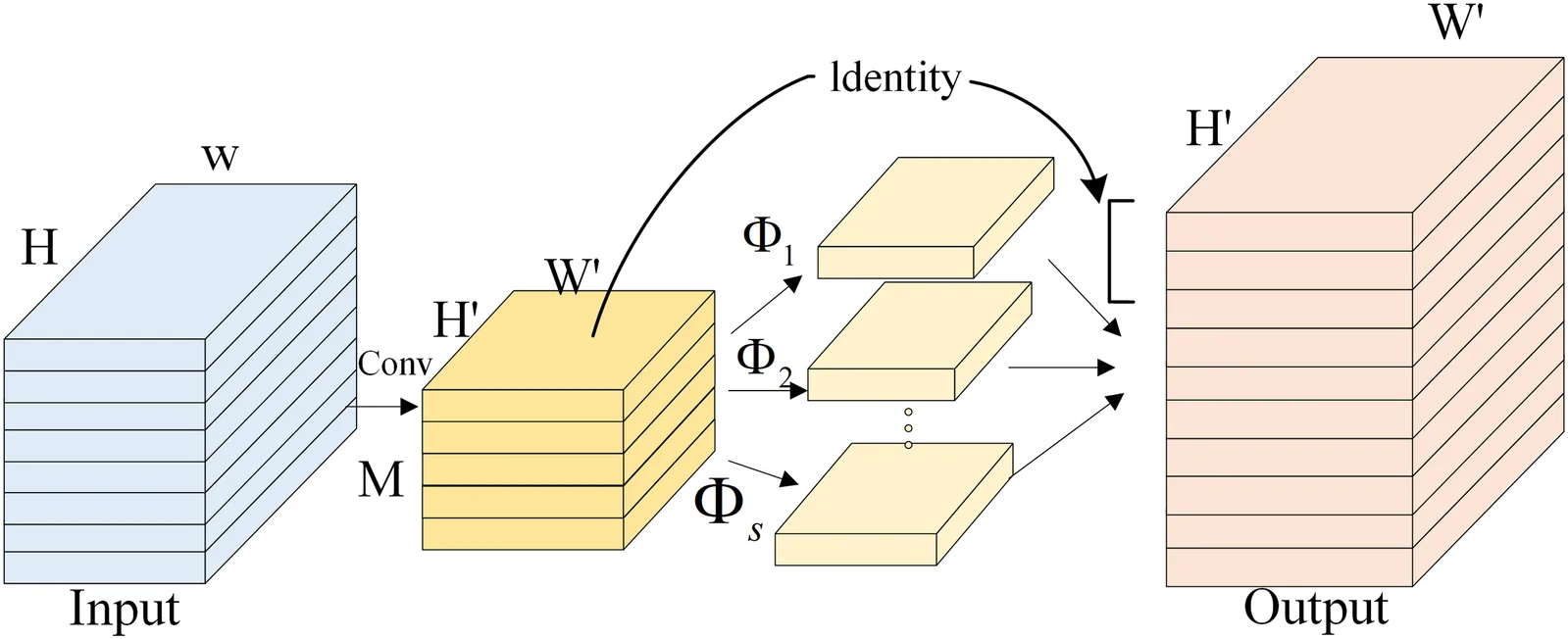
Ghost Module Structure.

The principle of Ghost Module is shown in the Fig 5. When processing an input feature map $𝒳\;\in {\mathbb{R}}^{h\times w\times c}$, h×w×c  where *hw* represents the spatial dimension and c denotes the number of channels, $f^{\prime }$ is related to the output dimension of the intrinsic feature map $w^{\prime }\times h^{\prime }\times m\;$, where *m* is the number of channels of the intrinsic feature map. Through the convolution operation ${𝒴}^{\prime }=𝒳*f^{\prime }$, the intrinsic feature map ${𝒴}^{\prime }$ is generated, achieving the preliminary and key information extraction of the input features. Subsequently, for the feature map ${𝒴}_{i}^{\prime }=(i=1,2,...,m)$ of each channel in the intrinsic feature map ${𝒴}^{\prime }$, a linear transformation operation $\Phi_{i,j}$ is introduced to generate Ghost feature maps. A single intrinsic channel generates *s* Ghost feature maps [38], which satisfies Eq. 3.


$$\begin{array}{c} y_{ij}=\Phi_{i,j}(y_{i^{\prime }})\;j=1,2,...,s\; \end{array}$$ (3)

Where $y_{ij}$ denotes the j−th Ghost feature map generated from the i−th intrinsic feature channel, $\Phi_{ij}(\cdot )$ represents a lightweight linear transformation (e.g., depthwise convolution), and *s* denotes the number of Ghost feature maps generated from each intrinsic feature channel. The final output feature maps are obtained by concatenating the intrinsic feature maps and all generated Ghost feature maps.

These transformation operations aim to mine the implicit redundant feature patterns in the intrinsic feature map with extremely low computational cost, expanding the diversity of feature expressions. Finally, through a concatenation operation, the intrinsic feature map and all generated Ghost feature maps are fused to obtain the final output feature map. By adopting the strategy of first extracting intrinsic features, then generating redundant Ghost features at low cost and fusing them, on the premise of ensuring the feature representation ability, compared with conventional convolution, it can effectively reduce parameters and computational complexity, adapting to the design requirements of lightweight networks.

Therefore, based on GhostModule, we designed the GDModule. The ordinary convolution module in GhostModule is replaced with DynamicConv. Since CNN can automatically adjust the weights of filters, we implement the linear mapping in GhostModule by using multiple Depthwise convs. This design enables the GDmodule to adaptively adjust the feature extraction strategy according to the input content. While maintaining the computational efficiency of GhostModule, it significantly enhances the model’s capability to represent complex patterns, achieving an effective balance between computational efficiency and feature expression ability.

**Fig 6:**
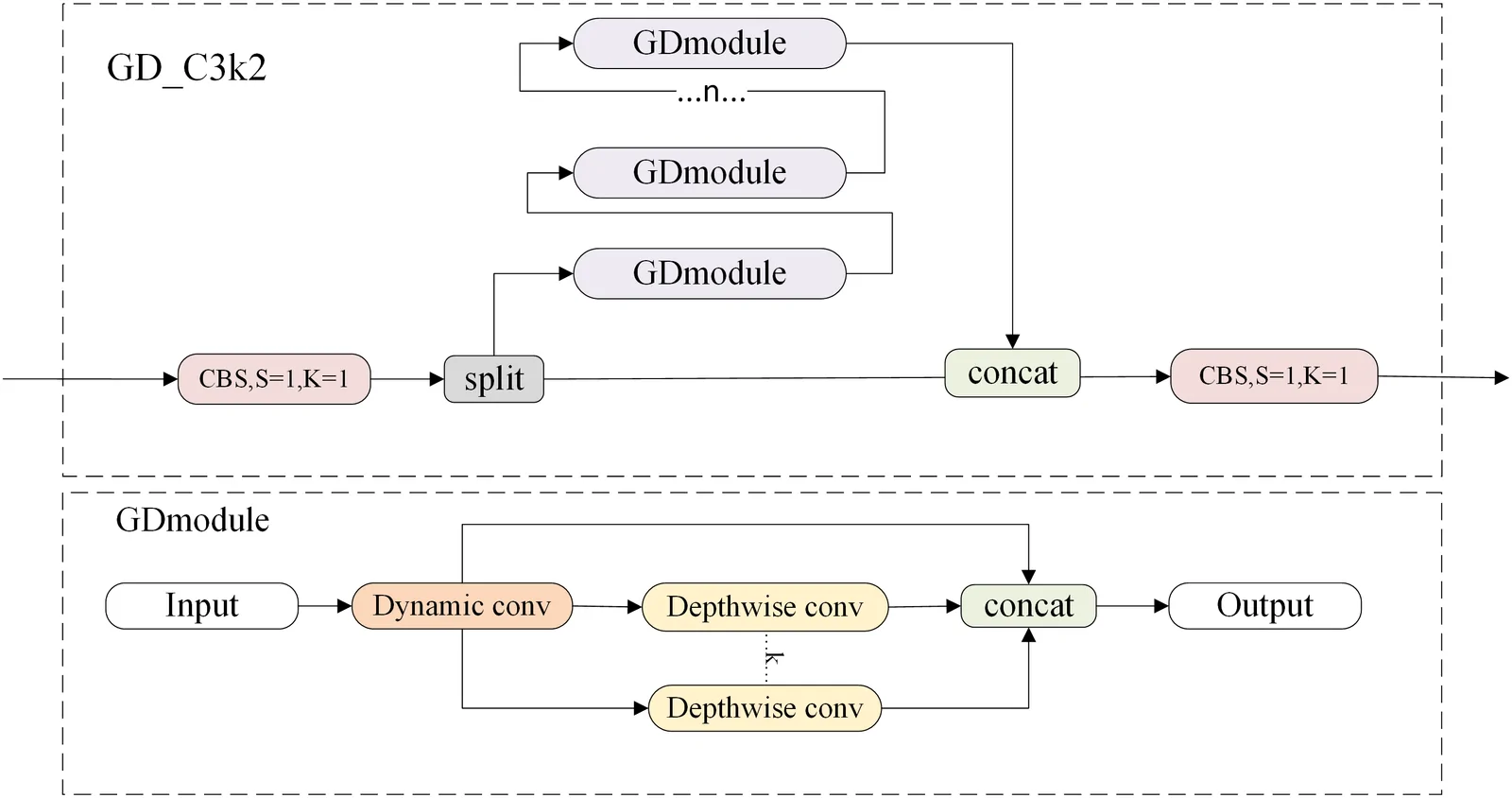
Structure diagram of GD_C3k2.

The structure diagram of GD_C3k2 is shown in Fig 6. This two-layer design, from the optimization of convolution operations inside the module to the reorganization of the overall structure, not only significantly improves the feature extraction accuracy of the network for small targets and occluded targets, but also effectively reduces the computational complexity and parameter quantity of the model through a dynamic computing mechanism. We achieve multi-dimensional performance improvement by co-designing GDmodule and GD_C3k2 components through a hierarchical module replacement strategy. Specifically, to address the problem that the static convolution kernels in the traditional GhostModule cannot adapt to the complex feature distribution of construction waste, we introduce DynamicConv, a convolution unit based on dynamic weight generation. This unit introduces a global context-aware module to perform statistical analysis on the input feature map in spatial and channel dimensions, generate adaptive weights, and use these to replace the conventional convolution in GhostModule, thus endowing the network with the ability to dynamically adjust convolution kernel parameters according to different scenarios. At the level of the GD_C3k2 network structure, we replace the traditional bottleneck layer in the original C3k2 structure with the self-designed GDmodule component, so as to optimize the residual connection and feature fusion of the original C3k2. GDmodule in the bottleneck can further enhance the network”s ability to extract multi-scale features of construction waste.

Compared with the original C3K2 module, the proposed GD_C3K2 introduces several structural and functional improvements that enhance feature representation capability while maintaining computational efficiency. In the original C3K2 structure, the bottleneck layers mainly rely on standard convolution operations with fixed kernel parameters to perform feature extraction and residual feature fusion. Although this design provides stable feature propagation, the static convolution kernels limit the adaptability of the network when facing complex feature distributions, such as irregular shapes, occlusion, and large intra-class variations commonly observed in construction waste detection scenarios.

To address this limitation, the proposed GD_C3K2 replaces the conventional bottleneck component in C3K2 with the GDmodule, which incorporates Dynamic Convolution to enable adaptive kernel generation. Through the global context-aware weighting mechanism, the GDmodule dynamically adjusts convolution kernel parameters according to the statistical characteristics of the input feature map. This dynamic computation mechanism allows the network to better capture diverse feature patterns and improves the flexibility of feature extraction compared with the fixed convolution operations used in the original C3K2.

Furthermore, the integration of GDmodule within the C3K2 framework optimizes the internal feature transformation and residual information flow of the module. By enhancing the interaction between dynamic feature extraction and residual feature fusion, GD_C3K2 improves the network”s ability to capture multi-scale structural characteristics of construction waste objects. This design is particularly beneficial for detecting small or partially occluded targets, where subtle feature variations play a critical role in accurate recognition.

Overall, while the original C3K2 focuses on efficient feature propagation using fixed convolution kernels, the proposed GD_C3K2 introduces dynamic feature extraction through GDmodule and Dynamic Convolution. This modification enables the network to achieve stronger feature adaptability and richer multi-scale representation capability, leading to improved detection performance while maintaining low computational complexity.

### 3.2 Integration of attention mechanism at neck-backbone junction

SimAM, as a parameter-free three-dimensional attention mechanism, draws its design inspiration from the spatial suppression effect in visual neuroscience. It aims to enhance the key features of convolutional neural networks by quantifying the importance of neurons in feature maps [39]. Introducing SimAM at the end of the backbone in YOLOv11 effectively addresses the problems of insufficient capture of key information and interference from background noise that exist in the feature extraction stage of traditional object detection models.

**Fig 7:**
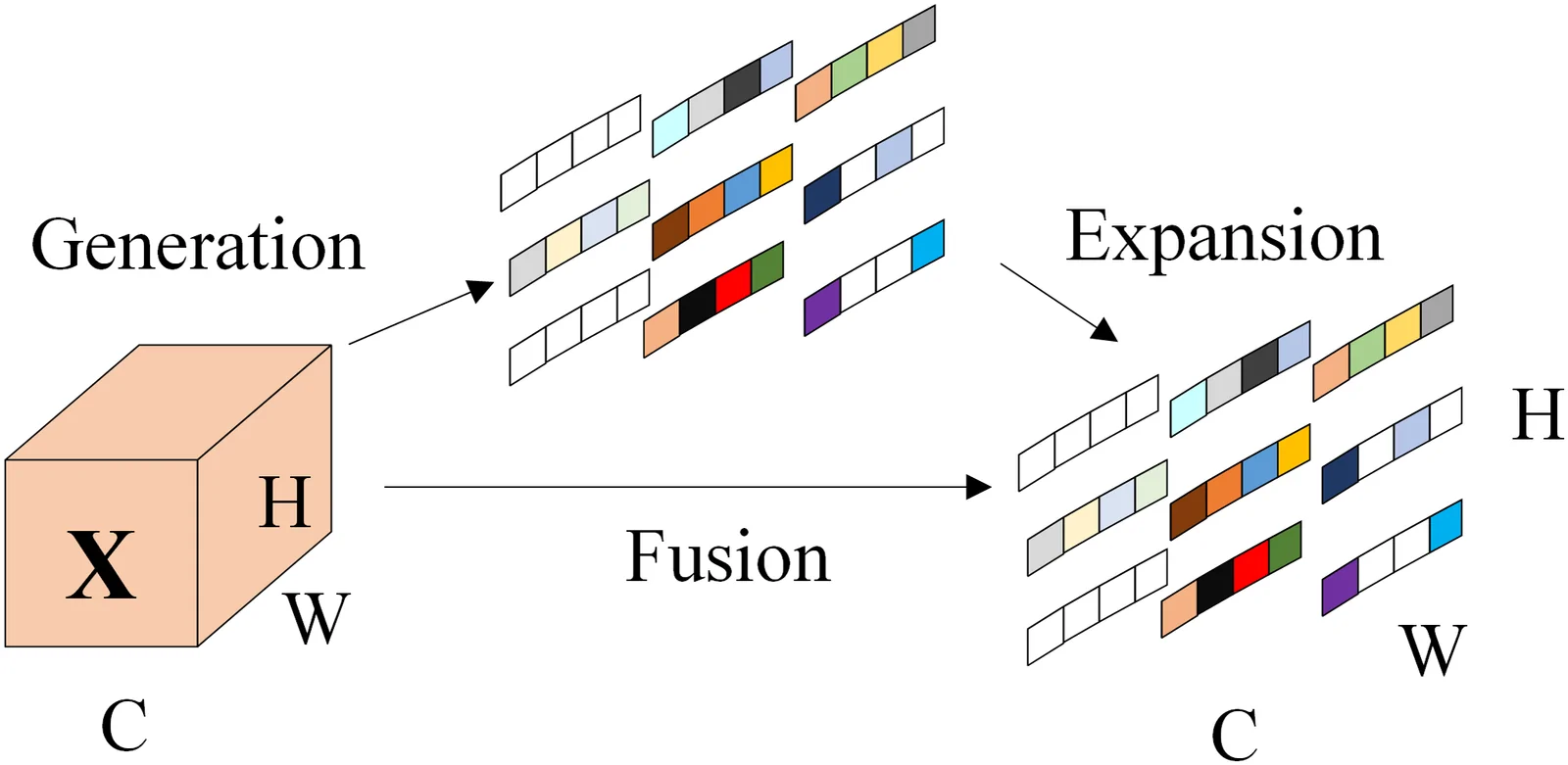
Structure of the SimAM attention mechanism.

The structure of the SimAM attention mechanism is illustrated in Fig 7. SimAM explores the relationships among neurons in a feature map and assigns an importance weight to each neuron. By reweighting the feature map, informative features are highlighted while irrelevant information is suppressed. Notably, SimAM does not introduce additional learnable parameters, which enables effective feature enhancement without increasing the model complexity. The computational process of SimAM is based on the construction and optimization of an energy function. The energy function is defined as:


$$\begin{array}{c} E(w,b)=\frac{1}{N-1}\sum_{j\neq i}(y_{j}-(wx_{j}+b){)}^{2}+(y_{i}-(wx_{i}+b){)}^{2}+\lambda w^{2} \end{array}$$ (4)

where $x_{i}$ denotes the feature value of the *i*-th neuron in the feature map, N represents the total number of neurons within the same channel, and $y_{i}$ is the binary label assigned to the neuron. Specifically, the target neuron is assigned $y_{i}=1$, while the remaining neurons are assigned $y_{j}=-1$. The parameters w and b denote the weight and bias of the linear classifier, respectively, and λ is a regularization coefficient used to prevent overfitting.

By minimizing the energy function with respect to w and *b*, the analytical solutions can be obtained as:


$$\begin{array}{c} w^{*}=\frac{x_{i}-\mu_{-i}}{\sigma_{-i}^{2}+\epsilon } \end{array}$$ (5)

$$\begin{array}{c} b^{*}=-w^{*}\mu_{-i} \end{array}$$ (6)

where $\mu_{-i}$ and $\sigma_{-i}^{2}$ denote the mean and variance of the neurons excluding the target neuron within the same channel, respectively, and ϵ is a small constant introduced to avoid numerical instability.

Substituting $w^{*}$ and $b^{*}$ into the energy function yields the minimum energy value:


$$\begin{array}{c} E_{min}=\frac{{\left(x_{i}-\mu \right)}^{2}}{4\left(\sigma^{2}+\epsilon \right)}+0.5 \end{array}$$ (7)

where μ and $\sigma^{2}$ represent the mean and variance of all neurons along the channel dimension. The importance of each neuron can be characterized by the inverse of the energy value, where a smaller energy corresponds to higher neuron importance. Finally, the sigmoid function is used to map the importance value to the attention weight:


$$\begin{array}{c} \alpha_{i}=\sigma \left(\frac{(x_{i}-\mu {)}^{2}}{4(\sigma^{2}+\epsilon )}+0.5\right) \end{array}$$ (8)

$$\begin{array}{c} Y=\alpha ⊙X \end{array}$$ (9)

where *X* denotes the input feature map, *Y* represents the enhanced feature map after applying attention, α denotes the attention weight map with the same dimension as *X*, and ⊙ represents element-wise multiplication. SimAM provides several advantages for feature enhancement. First, its parameter-free design avoids introducing additional learnable parameters, preventing an increase in model complexity and computational cost. Second, SimAM performs joint modeling across both spatial and channel dimensions of the feature map, enabling more comprehensive feature dependency modeling. Compared with traditional attention mechanisms such as SE and CBAM, which mainly focus on channel attention or adopt sequential attention modeling strategies, SimAM can capture feature importance more effectively.

The SimAM attention mechanism is introduced at the junction between the backbone and the neck of YOLOv11. At this stage, the backbone network has already extracted high-level semantic representations from the input image, while the neck is responsible for aggregating multi-scale features to support object detection at different spatial resolutions. Integrating SimAM at this transition stage allows the model to refine the extracted feature representations before they are propagated into the multi-scale feature fusion process, thereby improving the quality of the features used for subsequent detection.

Specifically, SimAM evaluates the importance of each neuron in the feature map by constructing an energy function derived from neural response theory. Through this mechanism, the contribution of each neuron to the overall feature representation can be quantitatively estimated, and adaptive weights are assigned to different spatial responses. Neurons that contain more discriminative information for object representation receive higher weights, whereas responses corresponding to redundant background patterns are suppressed. Unlike traditional attention mechanisms that operate only along channel or spatial dimensions, SimAM performs neuron-level modulation, enabling more fine-grained feature refinement across the entire feature map.

This property is particularly beneficial for detecting small construction waste objects. In practical construction-site environments, many waste materials such as steel bars, wood fragments, foam pieces, and small concrete debris often appear at relatively small scales in captured images due to large observation distances, irregular stacking patterns, and cluttered backgrounds. As a result, small targets typically occupy only a limited number of pixels and produce relatively weak feature responses in deep feature maps. During the hierarchical feature propagation and fusion process, these weak responses are easily dominated by stronger background activations, which may lead to missed detections or inaccurate localization.

By assigning higher importance weights to neurons that contribute more significantly to object representation, SimAM strengthens subtle but informative feature responses associated with small targets while suppressing irrelevant background activations. This neuron-level feature enhancement improves the signal-to-noise ratio of target-related information in the feature map. Consequently, the refined feature representations provide clearer and more distinguishable cues for the subsequent multi-scale feature fusion in the neck network, which ultimately improves the detection performance for small construction waste objects.

Moreover, SimAM is a parameter-free attention mechanism and does not introduce additional learnable parameters into the network. Therefore, the integration of SimAM does not significantly increase the computational complexity or model size of YOLOv11. This property ensures that the proposed method maintains the real-time inference capability of the original model while enhancing feature representation quality.

Overall, by refining feature responses before multi-scale fusion and enhancing weak target-related signals, the integration of SimAM effectively improves the detection capability of YOLOv11 for small construction waste objects without compromising computational efficiency.

### 3.3 Tahead

**Fig 8:**
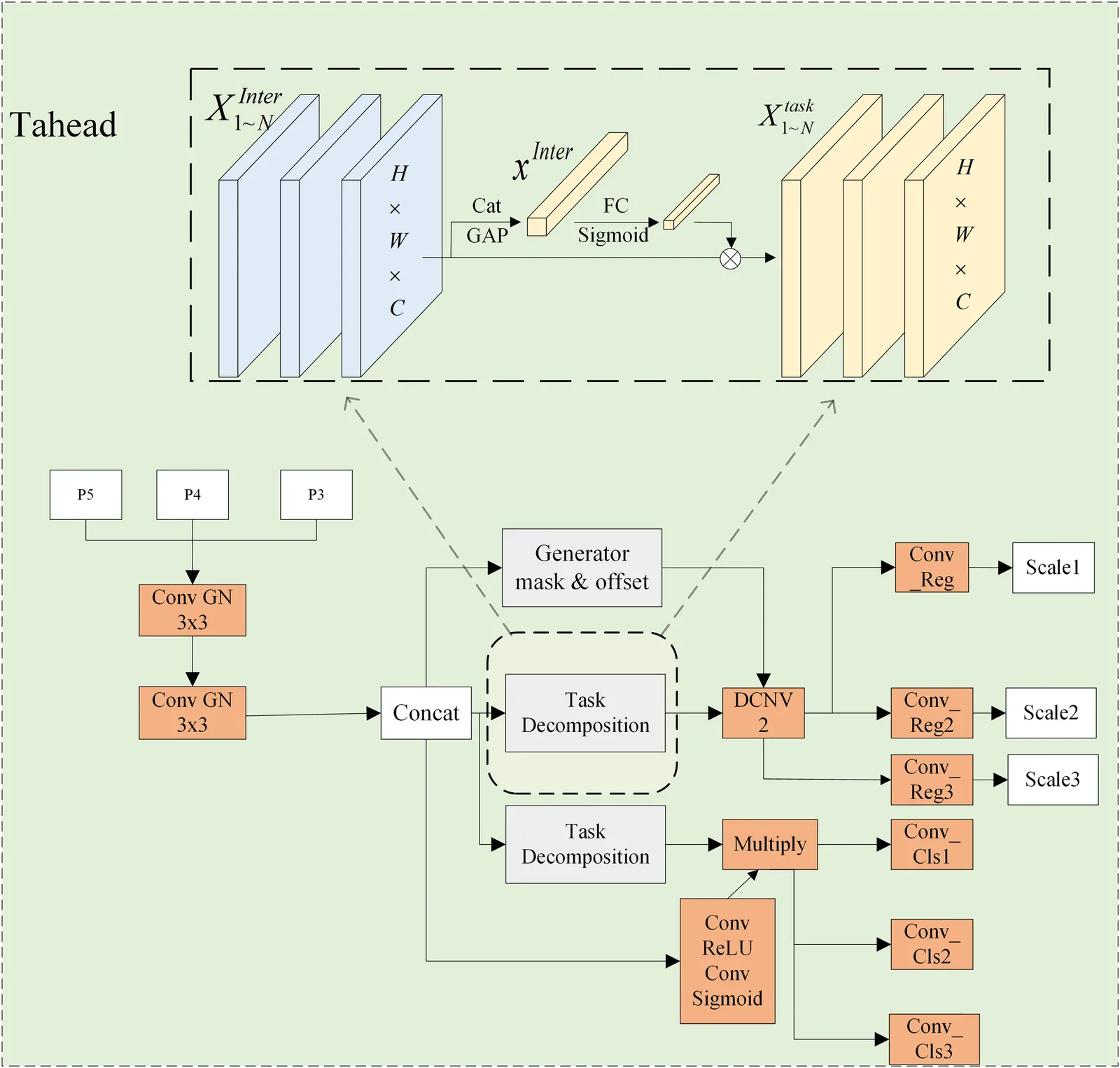
Structure diagram of the improved detection head Tahead.

In the field of object detection, the traditional YOLOv11 detection head suffers from problems such as insufficient feature fusion, task conflicts, and poor adaptability to geometric deformations. This study proposes an improved detection head design, whose structural diagram is shown in Fig 8. The core process is as follows: The improved detection head Tahead proposed in this study is based on TAP. Its core process is: The backbone network generates multi-scale features [*P*3, *P*4, *P*5], Each scale feature first undergoes dual-convolution and group normalization for preprocessing. For a single-level feature $P_{i}(i\in \{3,4,5\})$, As Eq. (10) execute.


$$\begin{array}{c} P_{i}=GN({Conv}_{3\times 3,C_{i}}(P_{i})) \end{array}$$ (10)

${Conv}_{3\times 3,C_{i}}$ is a 3x3 convolution with an output channel count of $C_{i}$, and GN stands for group normalization. After preprocessing, the features are concatenated along the channel dimension as $X_{concat}=Concat(P_{3},P_{4},P_{5})$. The concatenated feature $X_{concat}$ is fed into the dual-parallel Task Decomposition module. As a core module in the detection head architecture that bridges feature processing and accurate prediction, Task Decomposition decomposes complex detection tasks into sub-task streams adapted to classification and regression requirements upon multi-scale feature input. In the process, the fused features first flow into this module, and through internal mechanisms, core sub-tasks such as identifying construction waste categories and regressing target bounding box coordinates are learned separately, avoiding inter-task feature learning interference. In the mathematical workflow of this detection head module, multi-scale intermediate features $X_{1\sim N}^{inter}$ are first input, each with a dimension of H×W×C. For a single-level feature $X_{i}^{inter}(i\in [1,N])$, global average pooling is performed to extract the layer”s global statistical information, compressing the H×W×C tensor into a 1×1×C vector $x_{i}^{gap}$. Subsequently, via concatenation, multi-scale global information is fused as $x^{inter}=Cat\{x_{1}^{gap},x_{2}^{gap},...,x_{N}^{gap}\}$, yielding $x^{inter}$ with a dimension of 1×1×N×C as formulated in Eq. (11):


$$\begin{array}{c} x_{i}^{gap}=\frac{1}{H\times W}\sum_{h=1}^{H}\sum_{w=1}^{W}X_{i}^{inter}(h,w,:) \end{array}$$ (11)

Then, $x^{inter}$ undergoes a fully-connected transformation and is activated by Sigmoid to generate attention weights α; finally, the output attention is obtained according to Formula (12):


$$\begin{array}{c} X_{i}^{out}=\alpha_{i}\cdot X_{i}^{task},\;i\in [1,N] \end{array}$$ (12)

The attention weights are element-wise multiplied with the original task features $X_{1\sim N}^{task}$ layer by layer, and the weighted task features $X_{1\sim N}^{out}$ are output. This realizes the dynamic calibration of multi-scale features of construction waste, improving detection robustness, and enabling the model to more efficiently extract classification-discriminative features and regression-localization features. The decoupling is shown in Formula (13):


$$\begin{array}{c} \left\{X_{cls},X_{reg}\}=TaskDecomp(X_{concat}\right) \end{array}$$ (13)

Separating category semantics and spatial localization learning avoids task interference. To address the geometric deformations of construction waste, the regression branch introduces Dynamic Deformable Convolution (DCNv2). The task-decoupled $X_{reg}$ combines Δp and *M* generated by GeneratorMask & offset, following Eq. (14):


$$\begin{array}{c} X_{reg}^{dcn}=DCNv2(X_{reg},\Delta p,M) \end{array}$$ (14)

The dynamic adjustment of the convolution sampling position p yields the output as in Formula (15):


$$\begin{array}{c} X_{reg}^{dcn}(p)=\sum_{k=1}^{K}w_{k}\cdot X_{reg}(p+p_{k}+\Delta p_{k})\cdot M_{k} \end{array}$$ (15)

Here, $p_{k}$ are regular grid sampling points, $\Delta p_{k}$ denotes adaptive offsets, $w_{k}$ represents convolution weights, and $M_{k}$ is the attention mask value. The classification branch generates $\alpha_{cls}$ via Conv-ReLU-Conv-Sigmoid, and weights $X_{reg}^{dcn}$ as shown in Eq. (16):


$$\begin{array}{c} X_{reg}^{weighted}=\alpha_{cls}⊙X_{reg}^{dcn} \end{array}$$ (16)

During detection output: the classification branch uses Conv_Cls series convolutions, and category probabilities are predicted as $P_{cls}=Sigmoid(Conv\_Cls(X_{cls}))$; the regression branch uses Conv_Reg series convolutions to predict bounding box offsets. Combined with the decoding function, grid center coordinates $\left(x_{0},y_{0}\right)$, anchor dimensions $\left(w_{a},h_{a}\right)$, and scale-adaptive parameters $\left(s_{1},s_{2},s_{3}\right)$, the actual coordinates are transformed as in Eq. (17):


$$\begin{array}{c} B(x,y,w,h)=\left(x_{0}+\Delta x\cdot s_{i},\;y_{0}+\Delta y\cdot s_{i},\;w_{a}\cdot e^{\Delta w},\;h_{a}\cdot e^{\Delta h}\right) \end{array}$$ (17)

where Δx,Δy,Δw,Δh are offsets predicted by the regression branch, and $s_{i}$ is the scale-adaptive parameter for the *i*-th scale. Tahead enhances feature consistency via dual Conv-GN, alleviates task conflicts through Task Decomposition, adapts to irregular targets with DCNv2, strengthens key features via adaptive weighting, and optimizes detection performance for different sizes using multi-scale $s_{i}$. It mitigates conflicts between classification and regression tasks, achieving task-aligned functionality.

In conventional one-stage object detectors, the classification and localization tasks are typically performed on shared feature representations within the detection head. Although this shared structure improves computational efficiency, it often leads to a conflict between classification and regression objectives, since the two tasks emphasize different feature characteristics. Classification focuses more on semantic discrimination, while localization requires precise spatial and geometric information. This conflict may limit the detection performance, especially in complex scenarios such as construction waste detection where objects vary significantly in shape, texture, and scale.

To address this issue, the Task-aligned Head (TaHead) introduces a task decomposition strategy to decouple classification and regression features within the detection head. Multi-scale feature maps from the feature pyramid are first processed through convolutional layers to unify feature representations. These features are then concatenated and fed into the task decomposition module, which learns task-specific feature responses for classification and localization respectively.

Specifically, the task decomposition module aggregates intermediate features across different detection layers and performs global context modeling through global average pooling. The extracted global descriptor is further processed by a fully connected layer and a sigmoid activation function to generate adaptive task-aware weights. These weights are then used to reweight the intermediate feature maps, enabling the network to dynamically emphasize features that are more relevant to the specific detection task. Through this mechanism, TaHead effectively separates classification-sensitive features from localization-sensitive features, thereby reducing task interference and improving the representation capability of the detection head.

Furthermore, TaHead incorporates a dynamic convolution mechanism based on deformable convolution (DCNv2) to enhance spatial alignment between features and object regions. During object detection, the spatial distribution of construction waste is often irregular, and objects may exhibit complex shapes or partial occlusion. The deformable convolution layer generates adaptive offsets and modulation masks, allowing convolutional sampling positions to dynamically adjust according to the geometric structure of the target. This adaptive sampling strategy improves the alignment between convolutional receptive fields and object boundaries, thereby enhancing localization accuracy.

After task decomposition and spatial alignment, the refined features are processed by separate prediction branches for classification and regression. The regression branch predicts bounding box coordinates and scale information, while the classification branch estimates the probability distribution of different construction waste categories. By employing dedicated prediction branches for each task, TaHead further strengthens task-specific feature learning and improves the overall detection performance.

This design is particularly beneficial for detecting small construction waste objects. Small targets usually contain limited visual information and occupy only a small region in the feature map. When classification and localization tasks share the same feature space, subtle target-related features may be weakened during feature propagation. Through task decomposition, TaHead preserves task-relevant features and prevents important information from being diluted. Meanwhile, the deformable convolution module enhances spatial adaptability, enabling the detection head to better capture the structural characteristics of small and irregular waste objects.

Overall, TaHead improves detection performance by combining task decomposition and dynamic spatial alignment. By reducing task conflict and enhancing feature adaptability, the proposed detection head provides more discriminative and spatially aligned feature representations, which ultimately improves the detection accuracy of construction waste objects while maintaining efficient inference performance.

### 3.4 GTS-YOLO

**Fig 9:**
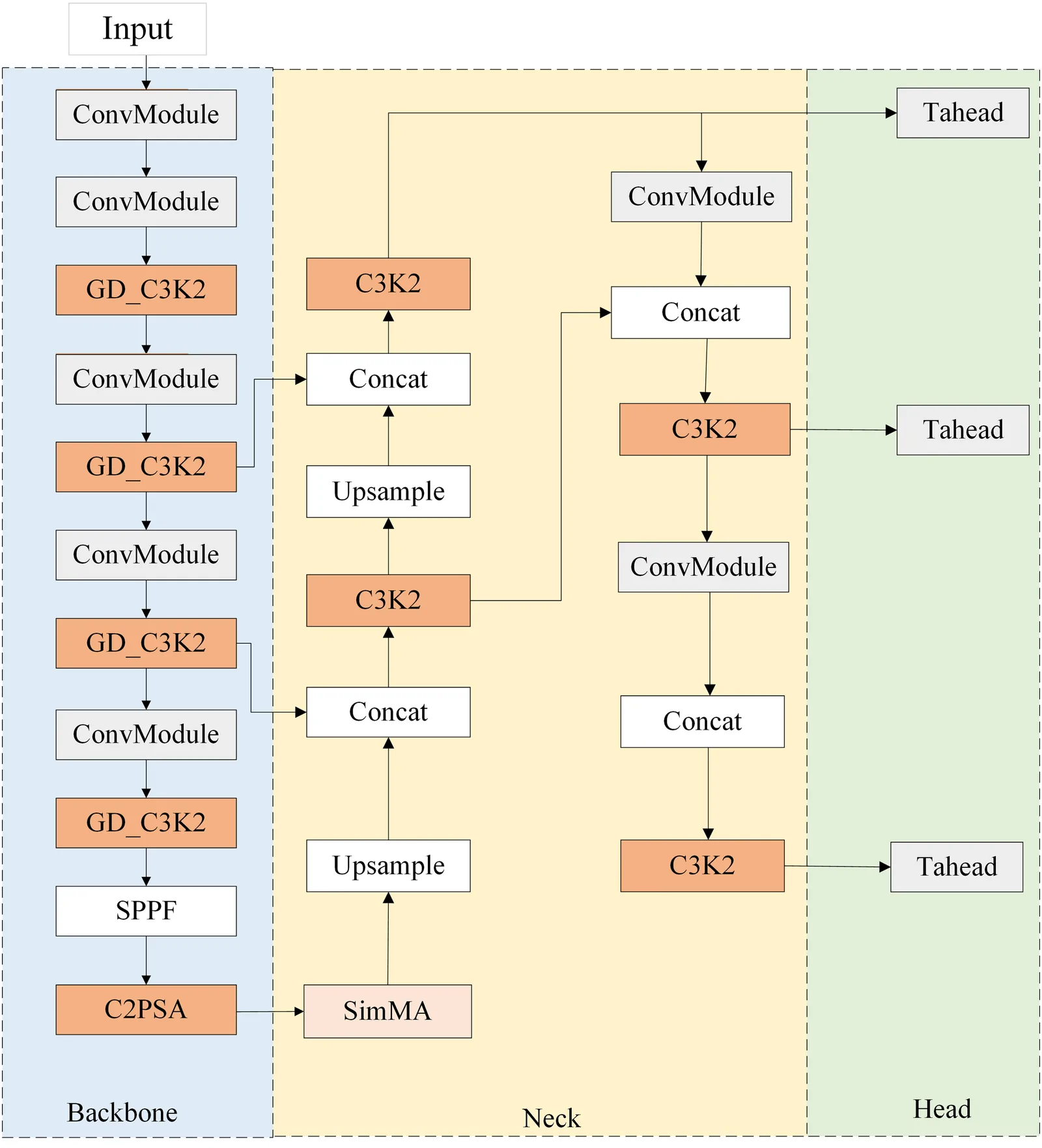
Overall architecture of the proposed GTS-YOLO model.

The overall architecture of the proposed model is illustrated in Fig 9, which follows the typical one-stage detection framework consisting of a backbone, a neck, and a detection head. The design aims to improve feature representation capability and detection accuracy for construction waste objects while maintaining the real-time performance of the YOLOv11 framework. To achieve this objective, several modules are introduced at different stages of the network, including the GD-C3K2 module in the backbone, the SimAM attention mechanism for feature enhancement, and the Task-aligned Head (TaHead) for optimized prediction. These components collaboratively enhance feature extraction, feature fusion, and prediction quality throughout the detection pipeline.

The backbone network is responsible for extracting hierarchical visual features from the input image. The backbone consists of multiple convolutional layers and GD-C3K2 modules that progressively transform the input image into high-level feature representations. The convolutional layers perform basic feature extraction and spatial downsampling, enabling the network to capture low-level patterns such as edges, textures, and structural information. Meanwhile, the GD-C3K2 modules further enhance feature representation by improving feature extraction efficiency while maintaining lightweight computation. Through this hierarchical structure, the backbone generates multi-level feature maps that contain both detailed spatial information and high-level semantic cues.

To further strengthen feature representation, the SimAM attention mechanism is introduced after the backbone feature extraction stage. SimAM operates by evaluating the importance of each neuron within the feature map using an energy-based formulation and assigning adaptive weights to different responses. This mechanism refines the extracted features by enhancing neurons that carry discriminative object information while suppressing irrelevant background activations. As a result, the feature maps entering the neck network contain more informative and noise-suppressed representations, which provides a stronger foundation for subsequent multi-scale feature fusion.

Following the backbone, the neck network performs multi-scale feature aggregation to enable robust detection of objects with varying sizes. As shown in the architecture, the neck adopts a feature pyramid structure that integrates features from different stages of the backbone through upsampling and concatenation operations. High-resolution shallow features provide precise spatial details, while deeper feature maps contribute stronger semantic information. By combining these complementary features, the neck effectively constructs multi-scale representations that are suitable for detecting both large and small construction waste objects. The C3K2 modules within the neck further refine the fused features, ensuring that the aggregated representations remain discriminative and computationally efficient.

After multi-scale feature fusion, the refined feature maps are passed to the detection head, where TaHead is employed to perform the final prediction. Unlike conventional detection heads that share features for classification and regression tasks, TaHead introduces a task decomposition mechanism that explicitly separates the feature learning process for classification and localization. This design alleviates the conflict between the two tasks and allows the network to learn task-specific representations. In addition, TaHead incorporates deformable convolution operations to dynamically adjust sampling positions according to object geometry, improving spatial alignment between features and target regions.

Through this design, the proposed architecture forms a progressive feature processing pipeline. The backbone extracts hierarchical features, SimAM enhances feature quality by emphasizing informative responses, the neck aggregates multi-scale representations to capture objects of different sizes, and TaHead performs task-aligned prediction to generate accurate detection results. Each component contributes to a different stage of the detection process, and their integration enables the model to achieve improved feature representation and prediction capability.

This architecture is particularly effective for construction waste detection scenarios, where objects often exhibit irregular shapes, varying scales, and cluttered backgrounds. Small objects such as steel bars, foam fragments, and small debris may occupy only a limited number of pixels and produce weak feature responses. The combination of attention-based feature enhancement, multi-scale feature fusion, and task-aligned prediction enables the model to preserve subtle target-related information and improve the distinguishability of small objects. Consequently, the proposed model achieves improved detection accuracy while maintaining efficient inference speed.

Overall, the proposed framework enhances the detection pipeline of YOLOv11 by integrating feature enhancement, multi-scale fusion, and task-aligned prediction mechanisms. Through the collaborative interaction of these modules, the model achieves stronger feature representation capability and improved detection performance for complex construction waste scenarios.

## 4 Experiments

### 4.1 Overview of the dataset

On this basis, we collected images of ten common types of construction waste. The dataset includes ten categories: steel bars, concrete, gravel, foam, bricks, asphalt, concrete fragments, tiles, scaffolding, and wood pieces. We recorded a total of 500 JPG images with a resolution of 1920×1200×3, and all samples were collected from a construction site in Xiangyang. The dataset was partitioned into training, validation, and test sets at an 8:1:1 ratio.

Field site access: No specific permits were required for field site access, as the study did not involve restricted areas, protected resources, or human subjects.

Ethical approval and informed consent: This study used both self-collected and publicly available datasets. All data were collected and used in accordance with ethical standards, and no personally identifiable information was involved. Clinical trial number: not applicable.

**Fig 10:**
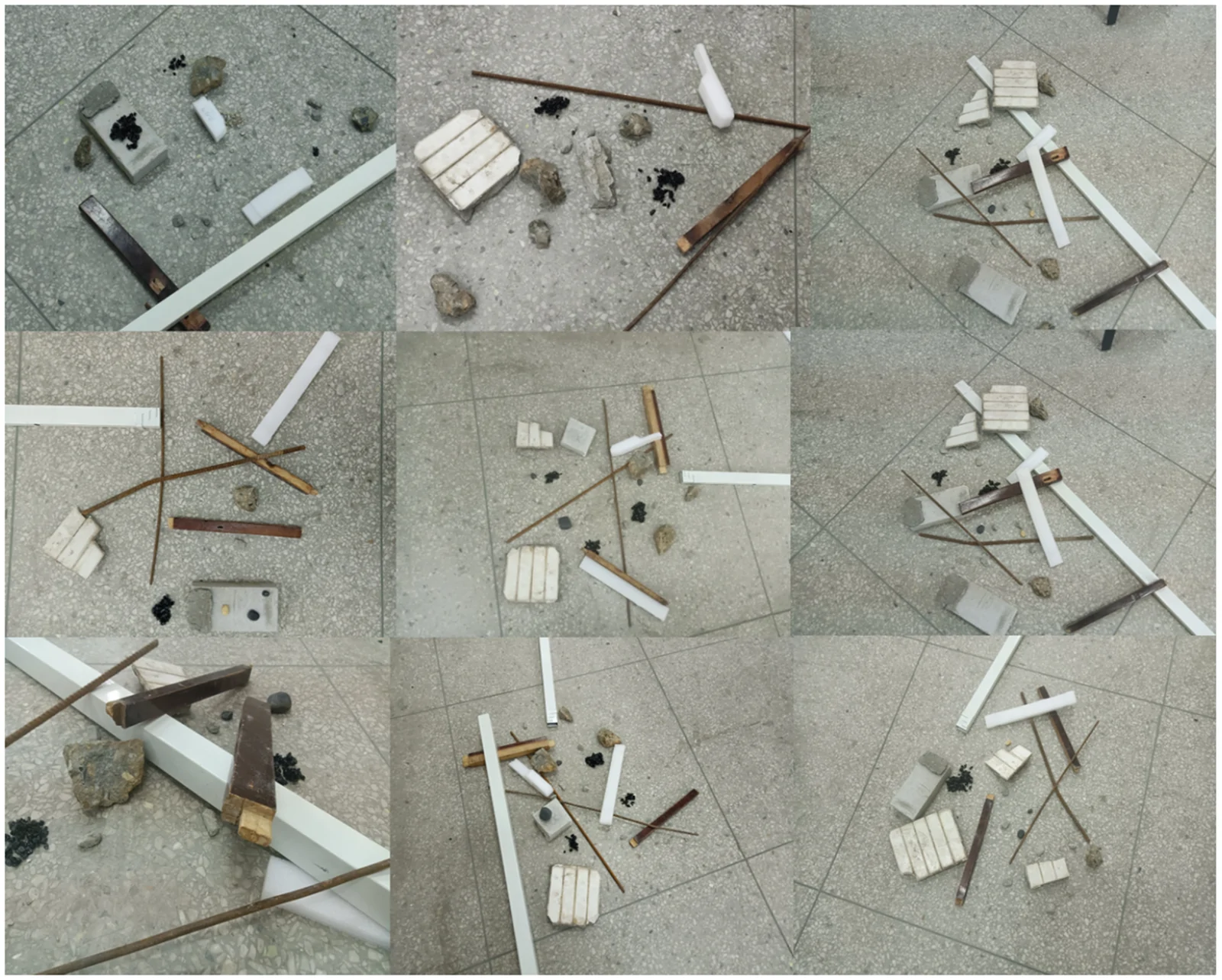
Samples of the dataset.

Examples of dataset samples (as shown in Fig 10) cover various complex scenarios, including both scattered and overlapping distributions. Each image contains both overlapping and discrete waste arrangements to simulate real-world construction waste distribution patterns.

**Fig 11:**
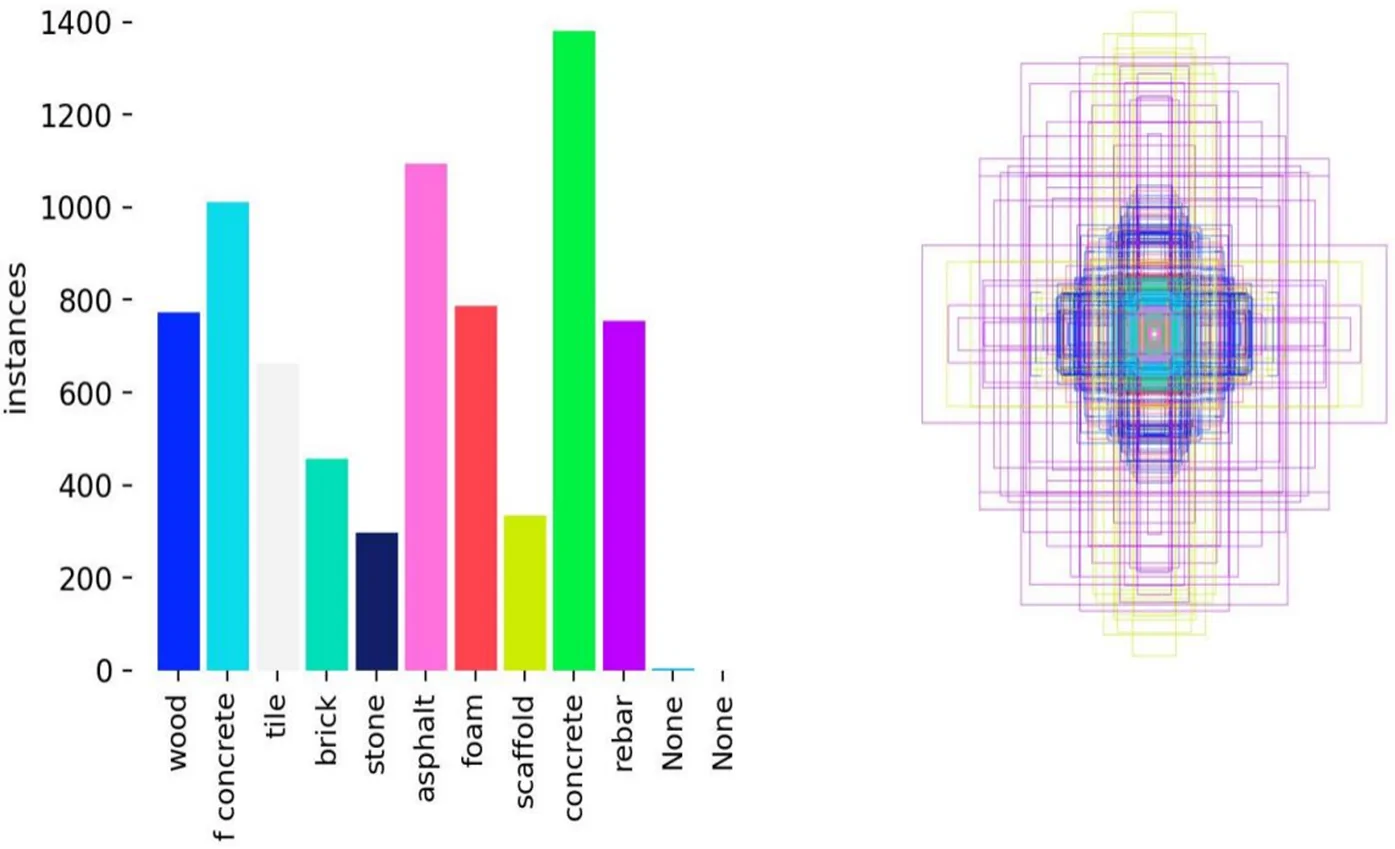
Distribution of sample categories.

The distribution of sample categories is illustrated in Fig 11. Specifically: wood pieces account for ~800 instances; fiber-reinforced concrete has ~1000 instances; tiles contain ~700 instances; bricks have ~400 instances; stone includes ~300 instances; asphalt has ~900 instances; foam contains ~300 instances; scaffolding has ~300 instances; concrete (the most abundant) has ~1400 instances; and steel bars account for ~800 instances. The total number of instances in the dataset is approximately 6,900.

In the contour plot, feature boundaries of different waste categories are represented by lines. Overlapping line regions reveal inter-category feature intersections (e.g., scaffolding and steel bars may share similar features, causing contour overlap). In contrast, separated regions indicate key feature intervals for category differentiation (e.g., the high-hardness feature of concrete and the softness of foam are completely separated in the contour plot).

### 4.2 Experimental configuration

The experimental setup was configured with a batch size of 32, 8 workers, 200 epochs, and an input image size of 640×640, while all other settings remained default. The detailed configuration is shown in Table 1. To ensure the reliability of the results, all experiments were repeated three times with independent random seeds, and the reported values represent the mean of these three runs. The variation across repetitions was minimal, with the standard deviation not exceeding ±1% for all reported metrics.

**Table 1:** Experimental Configuration table.

Item	Software Version
Operating System	Windows11
CPU	AMD r9 7945hx
GPU	NVIDIA GeForce RTX 4060 Laptop
Memory	8 GB
Acceleration Environment	CUDA v11.8
PyTorch Version	2.3.0
Python Version	3.9.19

### 4.3 Evaluation standards

In this experiment, Precision (P), Recall (R), mean Average Precision (mAP), F1-Score and FLOPs and parameters (params, reflecting model complexity) are adopted as evaluation metrics to assess the detection performance of construction waste. Precision (P) represents the proportion of correctly predicted positive samples, as formulated below.


$$\begin{array}{c} P=\frac{TP}{TP+FP}\times 100\% \end{array}$$ (18)

Recall (R) represents the proportion of positive samples in the dataset that are correctly predicted, as shown in Eq. (19).


$$\begin{array}{c} R=\frac{TP}{TP+FN}\times 100\% \end{array}$$ (19)

In the above two equations: for each category of construction waste, FP denotes the number of false positives, TP denotes the number of correctly detected instances, and FN denotes the number of false negatives.

mAP refers to the mean of the Average Precision (AP) across all categories. mAP is calculated at an IoU threshold of 0.5, also known as mAP@.5. A higher mAP indicates a better overall performance of the object detection network model. The calculation formula is as follows:


$$\begin{array}{c} mAP=\sum_{i=1}^{C}\frac{AP_{i}}{C} \end{array}$$ (20)

Additionally, mAP50-95 is a more comprehensive metric that evaluates model performance across multiple IoU thresholds. It is defined as the average of mAP values calculated at IoU thresholds from 0.5 to 0.95 with an interval of 0.05 (i.e., 0.5, 0.55, ..., 0.95), resulting in 10 thresholds in total. The calculation formula is:


$$\begin{array}{c} mAP50-95=\frac{1}{10}\Sigma k=110AP@IoU=0.5+0.05k \end{array}$$ (21)

where *k* ranges from 0 to 9, corresponding to the 10 IoU thresholds. This metric provides a holistic assessment of the model’s ability to maintain detection accuracy across varying levels of localization precision, making it more stringent than mAP@.5.

F1-Score is the harmonic mean of Precision (P) and Recall (R), which comprehensively reflects the detection performance by balancing the precision of predicted positive samples and the ability to retrieve all actual positive samples. Its formula is defined as:


$$\begin{array}{c} F1=2\frac{P\times R}{P+R} \end{array}$$ (22)

Model size refers to the physical storage space occupied by the model file, usually measured in megabytes (MB) or gigabytes (GB). The criteria for evaluating model lightweightness include the number of parameters (Params) and computational complexity (FLOPs), with their calculation formulas as follows:


$$\begin{array}{c} \text{params}=C_{o}\times \left(C_{i}\times K_{w}\times Kh+1\right) \end{array}$$ (23)

$$\begin{array}{c} \text{FLOPs}=\left[C_{i}\times K_{w}\times K_{h}+\left(C_{i}\times K_{w}\times K_{h}-1\right)+1\right]\times W\times H\times C_{o} \end{array}$$ (24)

### 4.4 Ablation experiment

To investigate the contribution of each proposed module to construction waste detection performance, ablation experiments were conducted using YOLOv11n as the baseline model. The proposed modules, including SimAM, GD_C3K2, and TaHead, were gradually integrated into the baseline network. The performance variations in terms of mAP50, Precision, Recall, F1-Score and parameter size were analyzed, and the results are presented in Table 2.

**Table 2:** Ablation Experiment Results.

Model	SimAM	GD_C3K2	Tahead	mAP50/%	Precision P/%	Recall/% R/%	F1-Score / %	Params/ M
YOLOv11n	×	×	×	64.23	66.91	66.14	66.52	2.58
2	✓	×	×	65.64	66.39	64.46	65.41	2.58
3	×	✓	×	65.06	67.12	65.01	66.05	2.28
4	×	×	✓	67.08	67.12	65.01	66.05	2.20
5	✓	✓	×	66.12	66.54	64.78	65.65	2.28
GTS-YOLO	✓	✓	✓	67.47	70.01	63.32	66.49	2.08

At the single-module level, different modules exhibit distinct performance characteristics. After introducing the SimAM attention mechanism, the mAP50 increases from 64.23% to 65.64%. This improvement can be attributed to the neuron-level attention weighting strategy of SimAM, which enhances discriminative feature responses and suppresses background interference. To further investigate the effectiveness of SimAM on small-object detection, category-level AP values were analyzed. In our dataset, stone, fragmented concrete (f concrete), and tile are typical small-scale targets because they usually appear as scattered fragments occupying limited pixels in the image. After introducing SimAM, the AP of stone increased from 32.7% to 37.0%, fragmented concrete increased from 72.0% to 78.0%, and tile increased from 90.7% to 92.5%. These improvements indicate that SimAM is able to enhance discriminative features of small construction waste objects while suppressing background interference. As a result, feature representations of small targets can be preserved more effectively during feature extraction and multi-scale fusion, leading to improved detection performance in complex scenes. However, the attention weighting mechanism of SimAM tends to focus more strongly on highly informative regions. Consequently, some heavily occluded or ambiguous targets may receive less attention during feature extraction, resulting in a slight decrease in Recall.

When the GD_C3K2 module is incorporated into the baseline network, the parameter size decreases from 2.58 M to 2.28 M while maintaining competitive detection accuracy (mAP50 of 65.06%). This improvement is mainly due to the dynamic convolution mechanism introduced in GD_C3K2, which enables adaptive kernel generation based on input features. Compared with static convolution kernels, this mechanism improves feature extraction efficiency while reducing redundant computations, thereby achieving a better balance between model lightweight design and detection performance.

In contrast, the TaHead module primarily improves detection performance by optimizing the prediction stage. By introducing task decomposition and deformable convolution mechanisms, TaHead enhances the alignment between feature representations and object regions, especially for multi-scale targets. As shown in Table 2, Integrating TaHead increases mAP50 to 67.08% and further improves Precision, indicating that the task-aligned prediction strategy effectively enhances both localization accuracy and classification reliability.

To comprehensively evaluate the model’s detection reliability, the F1-score was introduced as the harmonic mean of Precision and Recall (Table 2). The baseline YOLOv11n achieved an F1-score of 66.52% due to its highly balanced precision and recall. For the intermediate variants, individual integrations of SimAM, GD_C3K2, and Tahead yielded F1-scores of 65.41%, 66.05%, and 66.05%, respectively, reflecting minor trade-offs between false positives and missed detections.

Notably, our ultimate GTS-YOLO achieved a competitive F1-score of 66.49%. Although slightly lower than the baseline by 0.03% due to a restricted recall in highly cluttered environments, GTS-YOLO yielded the highest mAP50 (67.47%) and an optimal Precision of 70.01% (a 3.10% absolute improvement over YOLOv11n). This demonstrates that GTS-YOLO significantly reduces misclassifications while maintaining a robust overall performance. Crucially, this advanced detection capability was realized alongside a 19.38% reduction in parameter size (from 2.58 M to 2.08 M), firmly validating its superiority and efficiency for edge-device deployment.

Parameter analysis further reveals that the reduction in model size is mainly attributed to GD_C3K2. Compared with the baseline YOLOv11n (2.58 M parameters), GD_C3K2 reduces the parameter count to 2.28 M while maintaining competitive detection performance. Combined with TaHead, the final GTS-YOLO contains only 2.08 M parameters, demonstrating the effectiveness of the proposed lightweight design.

Overall, the ablation study demonstrates that the three proposed modules contribute complementary improvements to the detection framework. SimAM enhances feature discrimination, particularly for small-scale construction waste objects; GD_C3K2 improves feature extraction efficiency while reducing model complexity; and TaHead strengthens task-aligned localization and classification. Through the joint optimization of feature representation, lightweight architecture design, and prediction refinement, GTS-YOLO achieves the best overall performance with a mAP50 of 67.47% while reducing the parameter count by 19.38%, validating its suitability for real-time construction waste detection on resource-constrained devices.

### 4.5 Comparison experiments with mainstream models

To comprehensively evaluate the performance of construction waste detection models, this study conducted a systematic comparative experiment involving mainstream object detection algorithms. The experiment included two-stage classic algorithms (Faster R-CNN), single-stage efficient algorithms (SSD), Transformer-based rt-detr series, lightweight Swin series, Mamba-YOLO-B, and YOLO family models. The adaptability of different architectures in this task was analyzed through core metrics such as mAP (at 50% IoU threshold), Precision, Recall, parameter count (Params), and computational complexity (GFLOPs), the results were shown in Table 3.

**Table 3:** Comparison Results with Mainstream Models.

Model	mAP50/%	Precision P/%	Recall/% R/%	Params/M	GFLOPs
Faster R-CNN	62.13	63.54	60.97	137.07	370
SSD	58.35	60.78	64.65	26.28	63.0
rt-detr-resnet50	50.90	49.14	55.25	42.0	125.7
rt-detr-L	50.26	51.74	52.32	32.8	108.0
Swin-S	63.75	65.12	67.46	50.8	8.7
Swin-T	62.45	64.78	66.94	28.0	4.5
Mamba-YOLO-B	63.31	65.12	64.12	20.5	44.5
YOLOv5n	63.76	68.72	60.88	2.50	7.1
YOLOv8n	62.09	66.99	64.31	3.01	8.1
YOLOv10n	61.94	61.83	62.94	2.30	6.5
YOLOv11n	64.23	66.91	66.14	2.58	6.3
YOLOv12n	63.66	67.64	63.95	2.56	6.3
GTS-YOLO	67.47	70.01	63.32	2.08	7.1

In terms of detection accuracy, the two-stage model Faster R-CNN, with its cascaded design of Region Proposal Network and classification-regression modules, achieved an mAP50 of 62.13%, demonstrating the advantages of traditional architectures in feature extraction and target localization. However, single-stage models, with their end-to-end fast inference capability, are more competitive in construction waste detection scenarios with strong real-time requirements: YOLOv11n (mAP50: 64.23%), Swin-S (63.75%), and YOLOv5n (63.76%) all outperformed Faster R-CNN, confirming the efficiency of single-stage architectures in processing dynamic scene data. Notably, the proposed GTS-YOLO achieved a significant breakthrough in detection performance in complex scenarios by integrating three lightweight modules: SimAM, GD_C3K2, and Tahead. The SimAM module enhances key feature extraction through a parameter-free 3D attention mechanism; the GD_C3K2 module improves feature representation efficiency through a lightweight structural design based on Ghost and depthwise convolution operations; and the Tahead module optimizes multi-scale feature fusion strategies. Through their synergistic effect, GTS-YOLO achieved an mAP50 of 67.47% and a Precision of 70.01%, showing significant accuracy advantages among all compared models, fully verifying the effectiveness of the multi-module collaborative optimization strategy in identifying features in complex scenarios.

In terms of balancing efficiency and lightweight performance, each model exhibited distinct characteristics. Swin-T showed advantages of lightweight architecture with 4.5 GFLOPs and 28.0M parameters, but its mAP50 of 62.45% indicated a significant sacrifice in precision, failing to meet the requirements for detection accuracy in practical applications. Among YOLO family models, YOLOv10n, with 2.30M parameters and 6.5 GFLOPs, has potential for lightweight deployment, but its mAP50 of 61.94% did not reach optimal comprehensive performance. In contrast, GTS-YOLO achieved a substantial improvement in detection accuracy while maintaining only 2.08M parameters (lower than most YOLO models, such as YOLOv5n with 2.50M) and 7.1 GFLOPs (comparable to YOLOv5n). This improvement can be attributed to the collaborative optimization of lightweight feature extraction, attention enhancement, and efficient detection head design.

The Transformer-based RT-DETR series (e.g., RT-DETR-ResNet50 with 42.0M parameters and 125.7 GFLOPs) required substantially higher computational cost while achieving only 50.90% mAP50 on the construction waste dataset. This result suggests that Transformer-based architectures may not be as effective as lightweight CNN-based detectors for this task..

To verify that the performance improvement was not caused by random initialization, YOLOv11n and GTS-YOLO were trained independently three times using different random seeds. The mean and standard deviation of the evaluation metrics were calculated, and a t-test was conducted. The obtained p-values (<0.05) indicate that the performance differences between YOLOv11n and GTS-YOLO are statistically significant and unlikely to result from random initialization alone (Table 4).

**Table 4:** Statistical Significance Analysis.

Metric	YOLOv11n	GTS-YOLO	p-value
mAP50 (%)	64.23 ± 0.28	67.47 ± 0.25	0.007
Precision (%)	66.91 ± 0.42	70.01 ± 0.35	0.018
Recall (%)	66.14 ± 0.36	63.32 ± 0.41	0.024

Overall, GTS-YOLO achieved the highest mAP50 (67.47%) and Precision (70.01%) among all compared methods while maintaining only 2.08 M parameters and 7.1 GFLOPs. Although its Recall was slightly lower than that of YOLOv11n, the improvement in Precision and mAP50 indicates a stronger ability to suppress false-positive detections and accurately localize targets. Furthermore, the low standard deviations and statistically significant p-values (p < 0.05) demonstrate the stability and robustness of the proposed method across different training runs. These results confirm that GTS-YOLO provides an effective balance between detection accuracy and deployment efficiency, making it suitable for real-time construction waste detection in resource-constrained environments.

### 4.6 Performance comparison of adding different attention mechanisms

Given that the junction between the neck network (Neck) and the backbone network (Backbone) serves as a critical hub for feature interaction and fusion, its performance directly affects the model’s processing capability for the multi-scale and high-occlusion characteristics of construction waste. In this experiment, YOLOv11n was used as the baseline model, and 10 representative mainstream attention mechanisms, namely BiLevelRouting, SimAM, Triplet, MPCA, SegaNext, DA, Window Attention, CAFM, AGCA, and ECA, were selected and incorporated. The gain effects of each mechanism on feature fusion efficiency and object detection performance were quantified.

As shown in Table 5, after introducing BiLevelRouting, the mAP50 increased to 64.80%, but the Precision dropped to 66.45% and the Recall fell to 63.08%, with the parameter count and computational complexity increasing to 2.85M and 6.6 GFLOPs, respectively. This indicates that although the complex hierarchical routing of this mechanism can theoretically optimize feature transmission, its poor adaptability to the feature distribution of construction waste introduces redundant computations, interfering with the model’s focus on effective features.

**Table 5:** Performance Comparison Results of Different Attention Mechanisms.

Model	mAP50/%	Precision P/%	Recall/% R/%	Params/M	GFLOPs
YOLOv11n	64.23	66.91	66.14	2.58	6.3
BiLevelRouting	64.80	66.45	63.08	2.85	6.6
SimAM	65.64	66.39	64.46	2.58	6.3
Triplet	64.70	70.58	62.02	2.48	6.3
SegNext	60.72	61.58	60.95	2.57	6.3
Dual attention	62.87	64.69	62.40	2.75	6.5
Window Attention	60.88	64.61	63.81	2.57	6.4
CAFM	63.10	71.56	58.31	2.83	6.5
AGCA	63.73	65.79	64.75	2.55	6.3
ECA	63.22	65.56	66.30	2.58	6.3

As a parameter-free three-dimensional attention mechanism, SimAM, after being embedded, increased the mAP50 to 65.64%, with slight improvements in Precision and Recall without introducing additional parameters or computational load. This verifies its ability to constrain the feature space in a lightweight manner. However, due to the lack of targeted optimization for small targets and occluded scenes of construction waste, its gain in core detection performance is limited.

The Triplet mechanism caused the Precision to surge to 70.58%, but the mAP50 dropped to 64.70% and the Recall fell to 62.02%. This reflects that it excessively focuses on extracting category-discriminative features, at the expense of target localization and recall capabilities, making it difficult to adapt to the requirement of recognizing the integrity of construction waste targets in detection tasks.

After introducing mechanisms such as SegaNext, Dual attention, and Window Attention, their mAP50 values were all lower than that of YOLOv11n (SegaNext only reached 60.72%), and they had obvious shortcomings in either Precision or Recall. This indicates that the feature interaction patterns of these mechanisms do not match the multi-scale and strong-interference scene characteristics of construction waste, failing to effectively enhance the expression of key features.

Although CAFM, AGCA, and ECA showed varying degrees of improvement in mAP50 (AGCA at 63.73%, ECA at 63.22%), they did not exceed the baseline performance. Only ECA slightly led in the Recall metric (66.30%), reflecting its certain capability in capturing global features.

In summary, there are differences in the adaptability of different attention mechanisms in construction waste detection tasks. Most mechanisms, due to structural or logical conflicts with scene characteristics, failed to effectively improve performance and even had negative impacts. The lightweight mechanism SimAM, although maintaining efficiency, still struggled to significantly improve detection accuracy.

### 4.7 Comparison of actual detection effects

**Fig 12:**
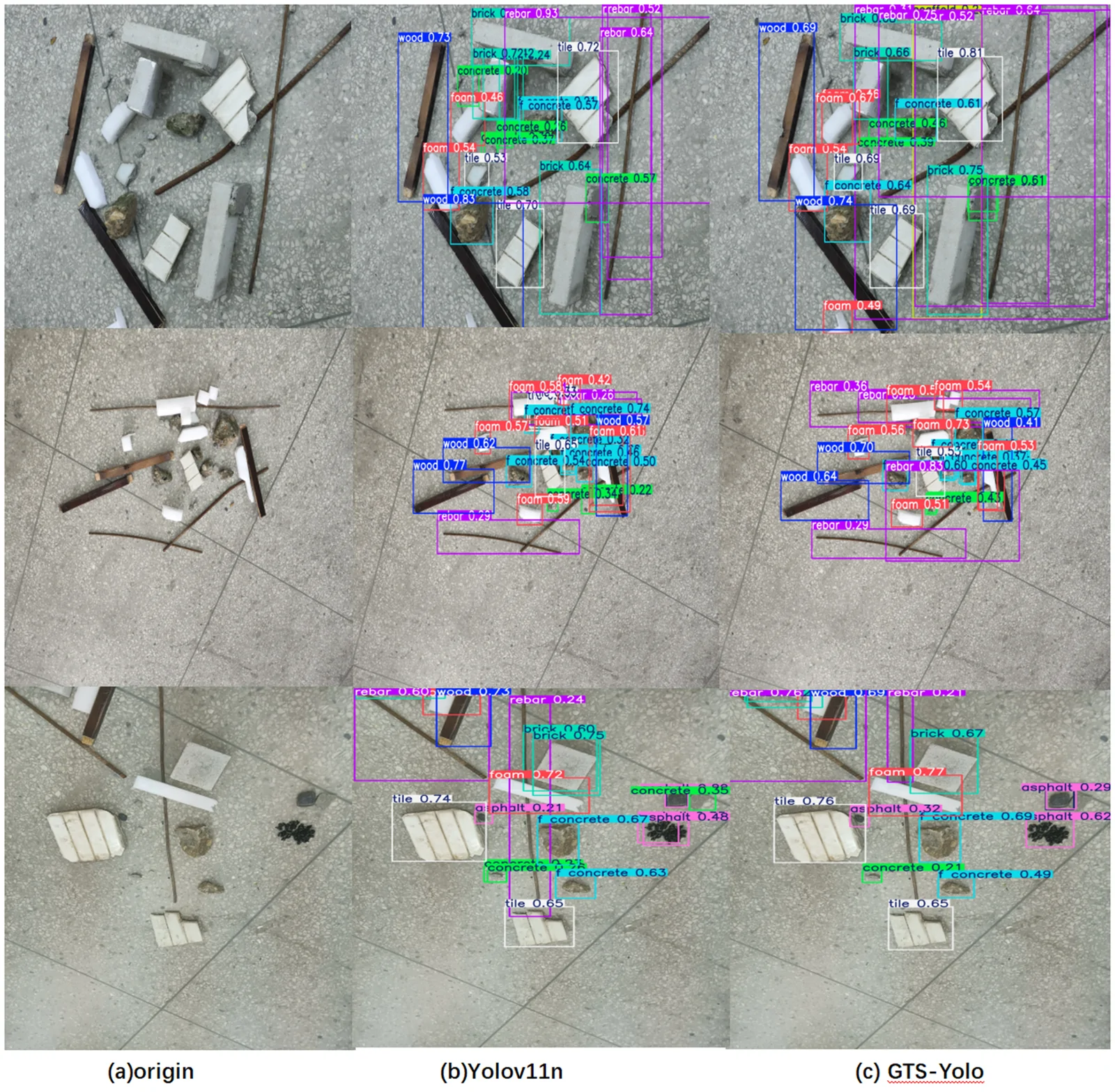
Comparison of Detection Results.

In the detection task of construction waste recognition, we conducted a comparative analysis of the detection results between YOLOv11n (column b) and GTS-YOLO (column c). Model performance can be comprehensively evaluated from Fig 12 based on bounding box fitting degree, and the occurrence of missed and false detections.

In terms of category labeling, YOLOv11n has obvious repeated detection issues. For example, in the middle image of the first row, the label “brick” is detected twice, reflecting defects in the model’s category prediction or output processing stages, which interferes with the interpretation of detection results. In contrast, GTS-YOLO displays category names such as “brick” and “tile” along with confidence values normally, without overlapping interference, showing better accuracy in category prediction.

Regarding bounding box fitting degree, some bounding boxes of YOLOv11n fail to accurately enclose object contours. As seen in the middle image of the first row, the bounding boxes for bricks and wood have problems such as “excessively large boxes” (including too much background) or “misaligned edges,” which affect positioning accuracy. GTS-YOLO”s bounding boxes, however, better fit the actual contours of objects. Whether for regular bricks and wood or irregularly shaped objects, they can tightly enclose targets, demonstrating more stable performance in positioning tasks.

In the comparison of missed and false detections, it can be observed from the original images (column a) that YOLOv11n has obvious missed detections. GTS-YOLO shows higher coverage in scenarios with small targets and stacked multiple objects, with fewer missed detections. The labeled target categories and quantities match the actual objects better, and false detections are effectively controlled.

In summary, from the intuitive analysis of visualized detection results, GTS-YOLO outperforms YOLOv11n in category labeling accuracy, bounding box fitting degree, and control of missed and false detections. It exhibits more ideal detection performance in the object detection task of construction waste recognition, providing a practical reference for model applications in subsequent.

### 4.8 Heatmap comparison and analysis

**Fig 13:**
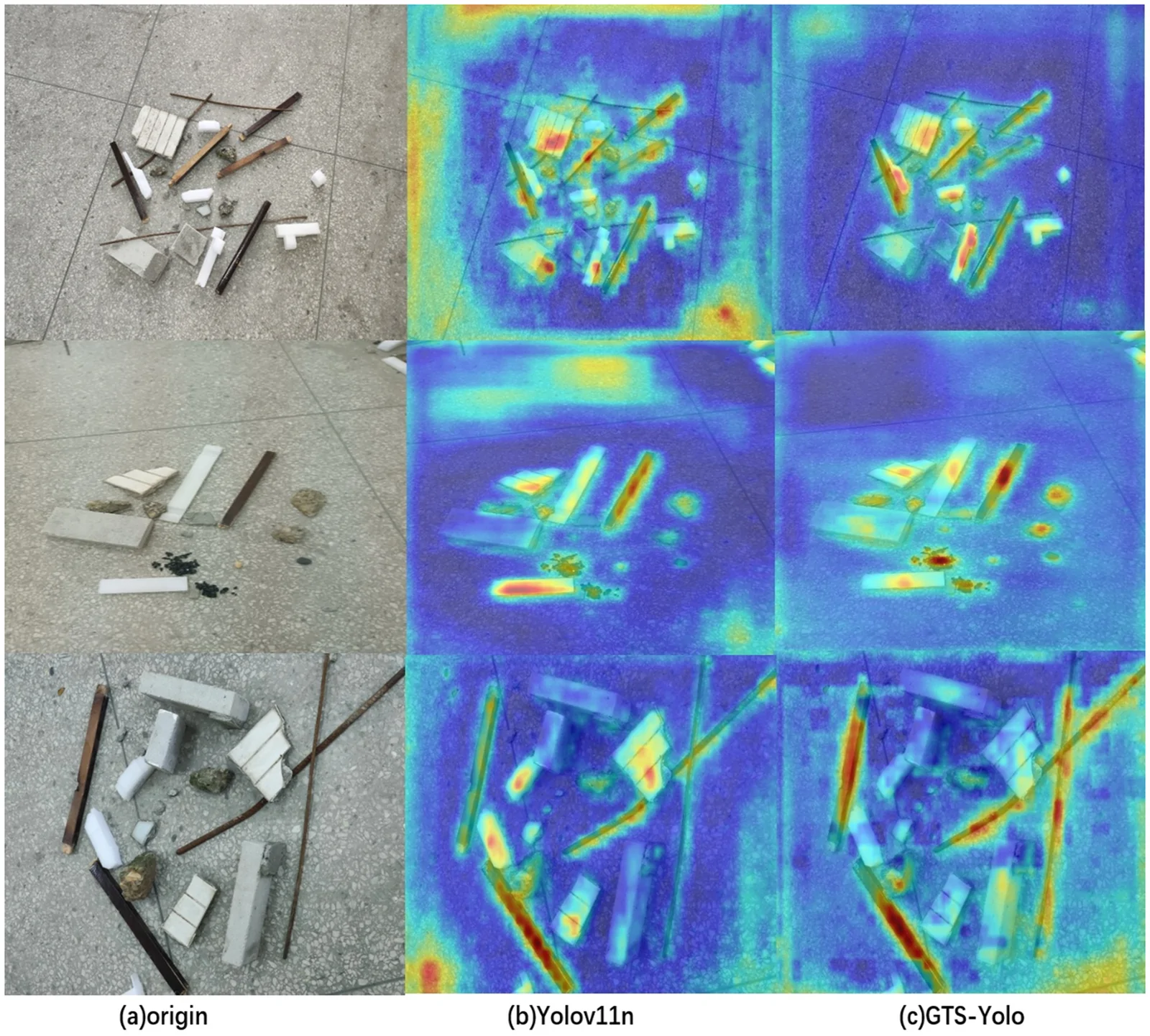
Heatmap Comparison and Analysis.

To conduct a systematic comparison between YOLOv11n and the improved GTS-YOLO model, we selected three representative images of construction waste piles as analysis samples, covering scenarios with background noise interference (such as construction site textures and non-target debris), occlusion by slender steel bars, and mutual coverage between objects. Based on Grad-CAM heatmap visualization technology, we analyzed the heatmap performance of the baseline model and GTS-YOLO. Specifically, in Fig 13 column (a) shows the original scenes, including complex working conditions such as irregular textures of cement ground, scattered gravel (background noise), steel bars occluded by bricks or wood, and mutual coverage of different types of construction waste; column (b) presents the feature response distribution of YOLOv11n; column (c) displays the feature response distribution of GTS-YOLO.

In terms of background noise suppression capability, the heatmap of YOLOv11n shows significant yellow responses in non-target areas such as cement cracks on the ground and scattered small stones, indicating that the model is prone to interference from background noise during feature extraction, leading to distracted attention. In contrast, the heatmap of GTS-YOLO only presents continuous red-yellow high responses in regions of real construction waste targets (e.g., bricks, wooden sticks, steel bars), while background regions are dominated by blue low responses. This verifies that through the synergy of dynamic convolution and attention mechanisms, GTS-YOLO can effectively filter background noise interference and accurately focus on target features.

For the challenging task of detecting occluded slender steel bars, in YOLOv11n”s heatmap, the part of the steel bar occluded by bricks or wood is almost completely covered by blue areas, with only sporadic yellow responses in the unoccluded segments. This fully exposes the limitation of traditional models in feature extraction when dealing with complex targets with “slender + occluded” characteristics. However, GTS-YOLO”s heatmap shows continuous yellow-red responses along the entire extension path of the steel bar, including the occluded part. This result indicates that the improved model, through the multi-scale feature fusion strategy, significantly enhances the ability to perceive the contour and locate the position of slender occluded targets.

In terms of detecting covered objects, in YOLOv11n”s heatmap, the response in regions of targets such as bricks and wooden sticks that are largely covered by other construction waste is fragmented and discontinuous, failing to effectively restore the complete contour of the objects. GTS-YOLO, relying on its contextual feature association mechanism, can utilize surrounding environmental information to complement missing features even when the target is completely occluded. In the heatmap, the region of the occluded object presents a clear and continuous high-response contour, reflecting its excellent feature reasoning and target integrity detection capabilities.

The mutual verification between the heatmap visualization analysis and quantitatively detection indicators (mAP50, Recall) fully demonstrates that GTS-YOLO significantly improves the model’s robustness to background noise, the detection accuracy of slender occluded targets, and the recognition integrity of covered objects in complex construction waste scenarios.

### 4.9 Loss analysis of training and validation processes

**Fig 14:**
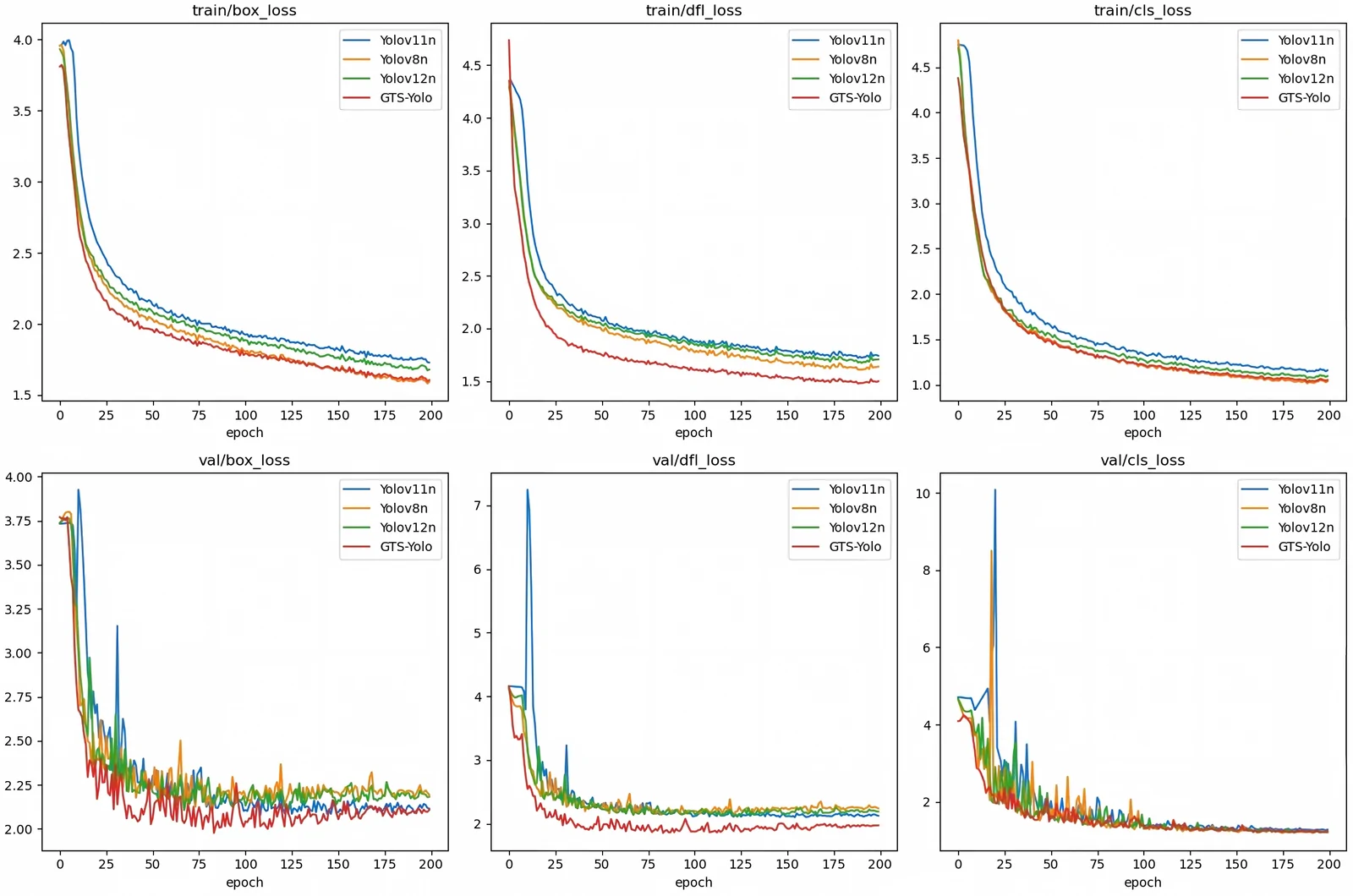
Loss Analysis of Training and Validation Processes.

As shown in Fig 14, The training and validation loss curves of the evaluated models reveal clear differences in convergence behavior and stability, which can be directly linked to the structural design of each network. Across all loss types—box regression loss, distribution focal loss (DFL), and classification loss—GTS-YOLO consistently demonstrates faster convergence and lower final loss values compared with YOLOv11n, YOLOv8n, and YOLOv12n.

Firstly, the box loss curves indicate that GTS-YOLO achieves more rapid reduction in localization error during the early epochs. This behavior can be attributed to the dynamic feature fusion module in the neck, which effectively aggregates multi-scale features, enhancing the network”s capacity to predict precise bounding boxes even in challenging contexts with varying object sizes. In contrast, YOLOv11n exhibits slower box loss reduction, reflecting its relatively simpler backbone and lack of targeted multi-scale feature enhancement.

Secondly, the DFL loss—responsible for accurate distribution prediction of bounding box offsets—shows that GTS-YOLO maintains a consistently lower training and validation loss throughout the epochs. This trend suggests that the attention-enhanced backbone in GTS-YOLO not only focuses on informative regions but also reduces interference from background features, facilitating more precise offset learning. YOLOv8n and YOLOv12n, lacking such attention mechanisms, converge more slowly and retain higher DFL loss, which correlates with their lower precision in detection results.

Thirdly, the classification loss curves reveal that GTS-YOLO achieves the fastest reduction and the most stable final loss. The improved classification performance is likely due to the combination of SimAM-based spatial attention and the enhanced feature representation in the backbone, which strengthens class discrimination, particularly in cases with overlapping or visually similar debris instances. YOLOv11n”s fluctuations in early epochs suggest less stable feature representation, leading to slower class separation.

Finally, the validation curves indicate that GTS-YOLO exhibits the least overfitting: the gap between training and validation loss is smaller across all three metrics. This stability can be linked to the lightweight yet structurally optimized modules, which reduce redundant parameters while preserving feature extraction capability. The regularized attention and dynamic fusion modules help the network generalize better, improving both precision and robustness.

In summary, the observed differences in loss convergence and final values can be directly interpreted through the structural innovations of GTS-YOLO. The attention mechanisms enhance discriminative feature learning, the dynamic fusion improves localization accuracy, and the careful balance of lightweight design with module optimization ensures stable training and strong generalization. These factors collectively explain why GTS-YOLO outperforms other lightweight YOLO variants in both detection metrics and training stability.

### 4.10 Testing on public datasets

The CODD dataset is a meticulously curated collection of images and annotations designed to support the development and benchmarking of both bounding box detection models and instance segmentation detection models for Construction and Demolition Waste (CDW) sorting. It encompasses 10 distinct categories of CDW, including bricks, concrete, tiles, wood, pipes, plastics, general waste, foaming insulation, stones, and plaster boards. The dataset comprises a total of 3,129 RGB images with a resolution of 1920×1200×3, containing 16,545 annotated samples, which are divided into 1,984 images for the training set, 570 images for the validation set, and 573 images for the test set.

The authors of the dataset have also released pre-trained weight files (all pretrained on the COCO dataset) for models in the YOLOv8n-l series. We conducted 100 epochs of training on the training set using all these weight files, along with GTS-YOLO and YOLOv11, and obtained the following data on the test set, the results were shown in Table 6.

**Table 6:** Public Dataset Testing Results.

Model	mAP50/%	mAP50-95/%	Precision P/%	Recall/% R/%	F1-score/%	Modelsize(MB)
YOLOv8n	42.62	40.39	44.64	39.57	41.96	5.97
YOLOv8s	44.42	43.26	45.55	40.92	43.12	11.9
YOLOv8m	45.05	44.23	46.50	47.95	47.21	25.8
YOLOv8l	45.45	44.59	48.67	41.32	44.70	43.7
YOLOv8x	45.01	44.08	47.81	39.69	43.38	68.2
YOLOv11n	43.11	41.54	45.94	42.32	44.06	5.33
GTS-YOLO	44.55	42.20	48.22	41.75	44.75	4.23

Among the evaluated models, YOLOv8l achieved the highest mAP50 (45.45%) and mAP50-95 (44.59%), followed closely by YOLOv8m (45.05% mAP50, 44.23% mAP50-95) and YOLOv8x (45.01% mAP50, 44.08% mAP50-95). Lighter models, including YOLOv8s, YOLOv8n, and YOLOv11n, exhibited lower mAP scores, reflecting the inherent trade-off between model complexity and feature representation capacity. Notably, the proposed GTS-YOLO achieved a competitive mAP50 of 44.55% and mAP50-95 of 42.20%, outperforming YOLOv8s, YOLOv8n, and YOLOv11n. This indicates that, despite a lightweight backbone, the integration of the attention-enhanced feature extraction and dynamic fusion modules effectively compensates for reduced model capacity, enabling robust detection performance.

Precision analysis reveals that GTS-YOLO reached 48.22%, ranking just below YOLOv8l (48.67%) and exceeding other variants significantly. This high precision can be directly attributed to the SimAM-based attention mechanism, which selectively emphasizes discriminative regions while suppressing irrelevant background features, thereby reducing false positives. In contrast, recall values suggest that while YOLOv8m leads (47.95%), GTS-YOLO maintains a competitive 41.75%, surpassing YOLOv8l (41.32%) and YOLOv8n (39.57%). This balance indicates that the dynamic feature fusion in the neck, which aggregates multi-scale features, enhances the network”s ability to detect diverse target instances without compromising precision.

The ablation study in Table 2 further supports this reasoning: each module—attention enhancement, dynamic fusion, and feature refinement—contributes incrementally to precision and mAP improvements. This layer-by-layer enhancement demonstrates that GTS-YOLO”s performance is not a coincidental outcome but a direct result of structural design choices. Evaluations on public datasets reinforce this conclusion, showing that the proposed modules improve both the discriminative power of features and the network”s sensitivity to small and overlapping targets, which are critical in CDW sorting tasks. Collectively, these results validate the design philosophy of GTS-YOLO: targeted module integration allows a lightweight network to achieve precision and robustness comparable to heavier models, illustrating that careful architectural design can mitigate the traditional trade-offs between efficiency and detection capability.

Despite the competitive performance of GTS-YOLO, it does not fully surpass certain benchmark models such as YOLOv8l and YOLOv8m. This outcome can be attributed to the inherent limitations of lightweight networks in terms of model capacity and feature representation. Larger models, including YOLOv8l and YOLOv8m, possess substantially higher numbers of parameters and computational resources, enabling them to capture more complex spatial and semantic features. In contrast, GTS-YOLO employs a lightweight backbone to reduce computation and memory cost, which inevitably constrains the overall expressive power of the extracted features.

The integration of attention-enhanced feature extraction (SimAM) and dynamic feature fusion effectively mitigates the reduction in model capacity, these modules primarily enhance local feature discrimination and small-target sensitivity. Consequently, while GTS-YOLO achieves high precision by suppressing false positives, its recall remains lower than that of larger models, reflecting a trade-off in covering all target instances, particularly under high IoU thresholds (mAP50–95).

Although the recall of GTS-YOLO (41.75%) is slightly lower than that of YOLOv11n (42.32%), the overall balance between precision and recall is improved. This can be verified by the F1-score, which increases from 44.06% to 44.75%. The improvement of F1-score indicates that the proposed method achieves a better trade-off between detection accuracy and robustness.

From the perspective of model efficiency, the proposed GTS-YOLO also shows clear advantages. The model size is only 4.23 MB, which is significantly smaller than YOLOv11n (5.33 MB) and other YOLOv8 variants. This reduction in model size demonstrates that the proposed network maintains competitive detection performance while effectively reducing model complexity, making it more suitable for deployment in resource-constrained environments. GTS-YOLO contains 327 convolutional layers and approximately 2.08 million learnable parameters, which is about 20% fewer than the baseline YOLOv11n. This reduction in parameters enables efficient deployment on resource-constrained devices without sacrificing detection performance.

This performance gap illustrates the classical trade-off between model complexity and detection capability: lightweight networks like GTS-YOLO can achieve competitive performance through targeted architectural enhancements, yet absolute performance remains constrained by the reduced parameter budget. Nevertheless, GTS-YOLO demonstrates that careful module design enables a lightweight network to approach the robustness and precision of heavier models, validating the proposed design philosophy for applications where efficiency and speed are critical.

## 5 Summary

In this paper, aiming at the problem of construction waste detection, improvements and experiments were carried out based on the YOLOv11 model. By adding lightweight convolution modules, integrating attention mechanisms, and improving the detection head, we proposed the GTS-YOLO model. Subsequently, we designed seven sets of experiments to verify the independent and comprehensive effects of various improvements: Through ablation experiments, key structures such as the GD_C3K2 module, SimAM attention module, and task-aligned detection head Tahead were gradually removed to analyze the independent contributions of each module in terms of accuracy, inference speed, and model complexity, so as to verify the rationality of their design. Secondly, mainstream object detection models such as YOLO series models, Swin-Transformer, and Mamba-YOLO were selected as control groups, and horizontal performance comparisons were conducted under the same experimental settings to evaluate the comprehensive performance of GTS-YOLO in terms of detection accuracy, inference efficiency, and parameter overhead. In addition, to explore the impact of attention mechanisms on feature extraction capabilities, we introduced mainstream lightweight attention modules and conducted replacement comparisons with the SimAM attention module under the same structure to analyze the performance differences of different mechanisms in complex backgrounds and small target recognition. combined with typical test images, the actual detection effects of different models in complex scenarios were compared, and the availability of the model in real applications was verified through visual detection boxes and analysis of missed and false detections. To intuitively understand the model’s focusing ability and discriminative mechanism, feature activation heatmaps were generated using the Grad-CAM method to compare the differences in the model’s attention to key regions under different structures. by tracking the changing trends of classification loss, localization loss, and total loss during training and validation, the performance of each model structure in terms of convergence speed, training stability, and optimal performance points was systematically analyzed. Finally, To further evaluate the generalization ability of the model, the performance of GTS-YOLO on the CODD construction waste detection dataset was tested.

In summary, the construction waste detection technology based on the GTS-YOLO model has certain value in lightweight construction waste detection and improving detection accuracy. Future research will continue to explore and improve in depth, providing more effective technical support for construction waste recycling.
